# An Adaptive Spiral Strategy Dung Beetle Optimization Algorithm: Research and Applications

**DOI:** 10.3390/biomimetics9090519

**Published:** 2024-08-29

**Authors:** Xiong Wang, Yi Zhang, Changbo Zheng, Shuwan Feng, Hui Yu, Bin Hu, Zihan Xie

**Affiliations:** 1 School of Information Science and Engineering, Yunnan University, Kunming 650091, China; 2 Inellifusion Pty Ltd., Melbourne 3000, Australia; 3 BEng Electrical and Electronic Engineering (EEE), Xi’an Jiaotong-Liverpool University, Suzhou 215123, China; 4 School of Information, University of Michigan, Ann Arbor, MI 48105, USA; 5 The School of Computer Engineering, Hubei University of Arts and Science, Xiangyang 441053, China; 6 Department of Computer Science and Technology, Kean University, Union, NJ 07083, USA; 7 Graduate Institute, Chinese Academy of Agricultural Sciences, Beijing 100091, China

**Keywords:** swarm intelligence, optimization algorithm, engineering design, adaptive strategy, unmanned aerial vehicles

## Abstract

The Dung Beetle Optimization (DBO) algorithm, a well-established swarm intelligence technique, has shown considerable promise in solving complex engineering design challenges. However, it is hampered by limitations such as suboptimal population initialization, sluggish search speeds, and restricted global exploration capabilities. To overcome these shortcomings, we propose an enhanced version termed Adaptive Spiral Strategy Dung Beetle Optimization (ADBO). Key enhancements include the application of the Gaussian Chaos strategy for a more effective population initialization, the integration of the Whale Spiral Search Strategy inspired by the Whale Optimization Algorithm, and the introduction of an adaptive weight factor to improve search efficiency and enhance global exploration capabilities. These improvements collectively elevate the performance of the DBO algorithm, significantly enhancing its ability to address intricate real-world problems. We evaluate the ADBO algorithm against a suite of benchmark algorithms using the CEC2017 test functions, demonstrating its superiority. Furthermore, we validate its effectiveness through applications in diverse engineering domains such as robot manipulator design, triangular linkage problems, and unmanned aerial vehicle (UAV) path planning, highlighting its impact on improving UAV safety and energy efficiency.

## 1. Introduction

The task of optimizing objective functions, whether through maximization or minimization within specified constraints, is pervasive across numerous fields. These fields include UAV route planning [1], image processing [2], mechanical system design [3], and social media sentiment analysis [4]. Consequently, optimization problems have consistently remained a critical and challenging focus of research.

In practical applications, many optimization challenges are often classified as “black-box” problems. These problems are characterized by the lack of explicit mathematical expressions, gradient information, and differentiability properties, making traditional optimization methods difficult to apply. In response to these challenges, swarm intelligence (SI) algorithms have gained popularity due to their simplicity and ease of implementation. These algorithms are inspired by the collective behavior observed in nature, such as the particle swarm optimization (PSO) [5], the Harris hawk optimization (HHO) [6], the dragonfly algorithm (DA) [7], and the Dung Beetle Optimization (DBO) [8].

Nevertheless, SI algorithms have their limitations, including slower convergence rates and lower convergence accuracy during the initial stages. Researchers have therefore proposed various enhancements to SI algorithms to improve their performance. This has led to the development of numerous optimization algorithms [9], including well-known metaheuristic algorithms like Genetic Algorithm (GA) [10], Differential Evolution (DE) [11], Grey Wolf Optimizer (GWO) [12], Whale Optimization Algorithm (WOA) [13], Butterfly Optimization Algorithm (BOA) [14], Sparrow Search Algorithm (SSA) [15], Chimp Optimization Algorithm (ChOA) [16], Arithmetic Optimization Algorithm (AOA) [17], and Artificial Jellyfish Search Optimizer (JS) [18]. Recent advancements in metaheuristic algorithms have also been applied in various scientific fields, including the inversion of self-potential anomalies, as demonstrated by Gobashy and Abdelazeem [19]. Their work highlights the broad applicability and continual evolution of metaheuristic methods in solving complex problems across different domains.

These metaheuristic optimization algorithms have shown superior performance compared to traditional optimization techniques in practical applications. They are valued for their robustness, ease of implementation, and excellent performance in solving complex optimization problems. However, much potential remains untapped in leveraging nature-inspired features, presenting opportunities for further advancements. Among these, the DBO algorithm, introduced by Xue J. et al. in 2023 [8], stands out as a novel swarm intelligence optimization algorithm inspired by the social behaviors of dung beetles. This algorithm categorizes the population into distinct roles, including rolling dung beetles, breeding beetles, small beetles, and stealing beetles.

Xue J. et al. [8] have successfully applied the DBO algorithm to various engineering design problems, demonstrating its practical application potential. Their experimental results indicate that the DBO algorithm effectively addresses real-world challenges, balancing global exploration and local exploitation with fast convergence rates and high solution accuracy.

Previous research has attempted to enhance the initialization randomness of the DBO algorithm using Q-learning [20] and antagonistic Q-learning [21]. However, these methods have shown drawbacks in practical applications, such as increased complexity and slower convergence speeds. Additionally, other researchers have used the sine–cosine search method to accelerate the DBO algorithm’s convergence [22], but this sometimes leads to the algorithm becoming trapped in local optima.

The No-Free Lunch (NFL) theorem [23] logically demonstrates that no single metaheuristic algorithm is universally applicable to all optimization problems. This theorem highlights that an algorithm’s performance is problem-dependent, meaning that a method excelling in one type of problem may perform poorly in another. Consequently, the NFL theorem has become a foundational principle in the field of optimization, driving researchers to continually explore and develop new metaheuristic algorithms tailored to specific problem scenarios. Given the diversity of real-world optimization problems, it is essential to continuously refine existing algorithms and introduce novel strategies to effectively address the unique challenges presented by each problem. While the DBO algorithm has shown significant optimization capabilities and fast convergence in many scenarios, it suffers from an imbalance between global exploration and local exploitation. This imbalance can cause the algorithm to become trapped in local optima, limiting its ability to thoroughly explore the solution space and potentially leading to suboptimal results. Furthermore, the DBO algorithm’s relatively weaker global exploration capability makes it less effective in finding the global optimum, especially in complex or high-dimensional problem spaces. These limitations underscore the importance of ongoing research and development in metaheuristics. By addressing these weaknesses, researchers can design enhanced algorithms that better balance exploration and exploitation, ultimately leading to more robust and versatile optimization tools. This continuous innovation is crucial for advancing the field and improving the ability to solve increasingly complex optimization problems across various domains.

While the DBO algorithm has demonstrated strong overall performance, it exhibits certain limitations that hinder its effectiveness. Specifically, during the reproduction phase, the algorithm tends to become trapped in local optima, especially near the origin (point 0). This often leads to the clustering of spawning DBOs at this point, which disrupts the algorithm’s iterative process and hinders its ability to explore other potential solutions. Additionally, the convergence factor in the DBO algorithm struggles to strike an effective balance between global exploration during the initial stages and local exploitation in the later stages. This imbalance ultimately reduces the accuracy of the solutions generated by the algorithm. These limitations highlight the need for further refinement and improvement, motivating the development of enhanced strategies that can address these challenges:

- The Gaussian Chaos strategy, a powerful method for population initialization, enhances various optimization algorithms by imparting the initial population with diversity, continuity, stability, and controllable parameters. These attributes significantly improve the search process efficiency, reduce the likelihood of local optima entrapment, and enhance global search capabilities, aiding in the discovery of superior solutions. This strategy is versatile, finding application in a wide range of optimization problems, especially complex static and dynamic scenarios. In our research, we have successfully integrated the Gaussian Chaos strategy into the DBO algorithm, further enhancing its performance.
- Inspired by the WOA, we have incorporated the Whale Spiral Search Strategy [24] into the DBO algorithm. This integration leverages whales’ remarkable navigational skills, characterized by intricate spiral patterns during hunting. By incorporating this approach into the DBO algorithm, we aim to enhance its exploration and exploitation capabilities, addressing existing limitations and improving performance in terms of convergence speed, solution quality, and global search efficiency. This integration underscores our commitment to advancing the DBO algorithm and pushing the boundaries of swarm intelligence optimization technology.
- We have introduced an adaptive weight factor into the DBO algorithm, resulting in several advantages. Firstly, it dynamically adjusts the weight factor, enhancing the algorithm’s search efficiency by better balancing global exploration and local exploitation, thereby improving the efficiency of finding optimal solutions. Secondly, this enhancement improves the algorithm’s robustness, making it more adaptable to various problem characteristics and reducing the likelihood of local optima convergence. Most importantly, this improvement boosts the algorithm’s global search capability, leading to more effective resolutions of complex optimization problems and increasing the likelihood of identifying the global optimum.

## 2. DBO

### 2.1. Rollerball Dung Beetle

In the wild, dung beetles encounter the challenge of maintaining a straight course while rolling their dung balls under the sun. Equation (1) from the original paper was used to update the position of the rolling dung beetle:

(1) $$\begin{array}{cc} \; & x_{i}\left(t+1\right)=x_{i}\left(t\right)+a\cdot k\cdot x_{i}\left(t-1\right)+b\cdot \Delta x\\ \; & \Delta x=\left|x_{i}\left(t\right)-X^{worst}\right| \end{array}$$

In the context of this study, *t* denotes the current iteration count, and $x_{i}\left(t\right)$ represents the position of the dung beetle at iteration *t*. The parameter *a* determines if the dung beetle deviates from its initial direction. It is probabilistically assigned a value of either 1 or −1, with 1 indicating no deviation and −1 indicating deviation. The parameter *k*, which ranges between (0, 0.2], represents the defect factor and was set to 0.1 in the original work. *b* is a constant value ranging between 0 and 1, and in this study, it was assigned a value of 0.3. $X^{worst}$ represents the global worst value, and Δ*x* is employed to mimic the influence of solar illumination, with a larger Δ*x* indicating that the dung beetle is farther from the light source.

In its natural habitat, when a dung beetle encounters an obstacle, it adjusts its rolling course by displaying behavior similar to a dance. To simulate this scenario, the original study introduced a probability-based method to determine whether the dung beetle encounters obstacles while rolling its dung ball. When encountering such an obstacle, a tangent function is used to determine a new rolling direction, mimicking the dance-like actions of the dung beetle. This process is described in the updated Equation (2), which determines the position of the rolling dung beetle:

(2) $$x_{i}\left(t+1\right)=x_{i}\left(t\right)+\tan\left(\theta \right)\left|x_{t}\left(t\right)-x_{i}\left(t-1\right)\right|$$

where θ⊆(0,π]; the position is not updated when $\theta =0,\frac{\pi }{2}$ and π.

### 2.2. Spawning Dung Beetles

In their natural habitat, dung beetles meticulously select a safe location for spawning. To mimic this behavior, the original paper presents a strategy for boundary selection to delineate this area, as outlined below:

(3) $$\begin{array}{cc} \; & Lb^{*}=\max\left(X^{best1}\times \left(1-R\right),Lb\right)\\ \; & Ub^{*}=\min\left(X^{best1}\times \left(1-R\right),Ub\right) \end{array}$$

Lb and Ub define the lower and upper bounds of the spawning area, while $X^{best1}$ represents the current local optimum. The parameter *R* is calculated as R=1−t/Tmax, where Tmax represents the maximum number of iterations. When the dung beetle identifies the optimal spawning region, it promptly spawns within it. According to the original text, each instance of spawning corresponds to a position update. The dynamic nature of the spawning region ensures a continued exploration of the vicinity housing the current best solution, thus preventing entrapment in a local optimum. The position update for the spawning dung beetle is governed by Equation (4):

(4) $$x_{i}\left(t+1\right)=X^{best1}+b_{1}\times \left(x_{i}\left(t\right)-Lb^{*}\right)+b_{2}\times \left(x_{i}\left(t\right)-Ub^{*}\right)$$

In the paper, $b_{1}$ and $b_{2}$ are random variables with dimensions of 1 × Dim, while Dim serves as an indicator for the optimization problem’s dimensionality.

### 2.3. Foraging Dung Beetles

In the natural environment, dung beetles engaged in foraging also exhibit behavior akin to selecting a secure location, much like when they lay eggs. The original text defines this area specifically using the following formula:

(5) $$\begin{array}{cc} \; & Lb^{b}=\max\left(X^{best2}\times \left(1-R\right),Lb\right)\\ \; & Ub^{b}=\min\left(X^{best2}\times \left(1-R\right),Ub\right) \end{array}$$

In this context, $X^{best2}$ stands for the best global position, $Lb^{b}$ and $Ub^{b}$ signify the lower and upper limits of the optimal foraging area, while Lb and Ub denote the lower and upper bounds for problem-solving. Each foraging action executed by the dung beetle correlates with one position update, and the position of the foraging dung beetle is modified as follows:

(6) $$x_{i}\left(t+1\right)=x_{i}\left(t\right)+C_{1}\times \left(x_{i}\left(t\right)-Lb^{b}\right)+C_{2}\times \left(x_{i}\left(t\right)-Ub^{b}\right)$$

$C_{1}$ is a random number following a normal distribution, and $C_{2}$ is a vector of size 1 × Dim, with its values falling within the range of [0, 1].

### 2.4. Stealing Dung Beetles

In nature, certain dung beetles choose to steal dung balls from other individuals of their species. To simulate this behavior, the original paper assigns the optimal global location Xb as the position of the contested dung ball. The act of stealing, carried out by the dung beetle involved in this behavior, results in a location update, as formulated by the following equation:

(7) $$x_{i}\left(t+1\right)=X^{best2}+S\cdot g\cdot \left(\left|x_{i}\left(t\right)-X^{best1}\right|+\left|x_{i}\left(t\right)-X^{best2}\right|\right)$$

As detailed in the original manuscript, *S* is defined as a constant with a fixed value of 0.5. The variable *g* corresponds to the size of a random variable, and Dim is utilized to denote the dimensionality of the problem under investigation. The initial text specifies the population sizes for various dung beetle categories as follows: six for rolling dung beetles, six for breeding dung beetles, seven for foraging dung beetles, and eleven for stealing dung beetles.

### 2.5. DBO Algorithm Implementation Steps

The pseudocode for the DBO algorithm is in Algorithm 1.
**Algorithm 1** Framework of the DBO Algorithm**Input:** Maximum iteration $T_{max}$, population size *N***Output:** Optimal position $X^{best2}$ and its corresponding fitness value $f_{\min}$1:Initialize the population of particles, indexed as i=1,2…N, and def-ine relevant parameters.2:**while** $t\leq T_{\max}$**do**3:   **for** *i* belonging to the rolling dung beetles group. **do**4:     a=rand(1)5:     **if** a≤0.9 **then**6:        Update the location of the rolling dung beetle using Equation (1).7:     **else**8:        Simulate rolling the ball in the presence of obstacles using Equation (2) to update the location.9:     **end if**10:   **end for**11:   Calculate the nonlinear convergence factor as $R=1-t/T_{max}$.12:   **for** *i* belonging to the spawning dung beetles group. **do**13:     Update the location of the spawning dung beetle using Equations (3) and (4).14:   **end for**15:   **for** *i* belonging to the foraging dung beetles group. **do**16:     Update the location of the foraging dung beetle using Equations (5) and (6).17:   **end for**18:   **for** *i* belonging to the stealing dung beetles group. **do**19:     Update the location of the stealing dung beetle using Equation (7).20:   **end for**21:**end while**22:**return** Return the optimal position $X^{best2}$ and its corresponding fitness valu-e $f_{\min}$.

### 2.6. The Time Complexity of DBO

The time complexity of the DBO algorithm can be determined by analyzing its key components. The initialization phase has a complexity of O(N), where *N* is the population size. The main loop runs for $T_{\max}$ iterations, and within each iteration, the updated operations for all groups of dung beetles (rolling, spawning, foraging, and stealing) have a combined complexity of O(N). Therefore, the overall time complexity of the DBO algorithm is $O(T_{\max}\cdot N)$.

## 3. Improving the Dung Beetle Optimization Algorithm (ADBO)

### 3.1. Motivation

The DBO algorithm demonstrates a superior convergence speed compared to traditional algorithms such as WOA and POS, and it surpasses other algorithms like SSA and HHO in attaining global optimum solutions. It maintains a relatively balanced performance in terms of both seeking global optimal solutions and convergence speed. However, achieving the ideal optimal solution remains an exceptionally challenging task for the DBO algorithm. Moreover, its capability to address complex problems is relatively weak. Despite its strengths, such as robust search abilities and fast convergence, the DBO algorithm exhibits an imbalance between global exploration and local exploitation, which makes it susceptible to local optima and limits its global exploration capabilities. Therefore, this chapter introduces three strategies to enhance the search performance of the DBO algorithm.

### 3.2. Initialize the Population Using Chaotic Mapping

Chaotic mapping is a method that combines determinism and randomness. Chaos exhibits characteristics such as randomness and aperiodicity [25]. During the initialization and updating processes, chaotic variables replace random variables. This enables chaotic mapping strategies to explore a wider range of the solution space compared to random search strategies. As a result, chaotic initialization significantly enhances the search breadth of optimization algorithms, particularly during the random initialization process.

The DBO algorithm involves the random initialization of population positions in the search space. However, this approach has three main disadvantages:

- Uneven distribution of dung beetle individuals’ positions.
- Limited global exploration capability.
- Low population diversity, making it susceptible to local optima.

To diversify the initial solutions within the population, the DBO algorithm integrates chaotic mapping during the initialization phase. This creates highly diversified initial populations. Various chaotic mapping techniques are available, including Singer mapping, Chebyshev mapping, Bernoulli mapping, Gaussian mapping, PWLCM mapping, and others [26,27,28]. Incorporating Gaussian Chaos into the DBO algorithm offers several academic advantages. Firstly, Gaussian Chaos generates random variables that closely approximate a normal distribution, enhancing the diversity of initial solutions during the population initialization phase of the DBO algorithm. Consequently, it facilitates a comprehensive exploration of the search space and mitigates the risk of being trapped in local optima. Secondly, Gaussian Chaos introduces a higher degree of controllable randomness, expediting the convergence of the DBO algorithm and leading to a quicker attainment of optimal solutions. Moreover, utilizing Gaussian Chaos enhances the algorithm’s stability, ensuring consistency and reproducibility across multiple executions. Lastly, the characteristics of Gaussian Chaos are particularly suitable for scenarios requiring reduced noise and increased controllability. However, it is imperative to carefully select parameters and chaos mapping methods when integrating Gaussian Chaos, as its influence can vary depending on the specific problem. Furthermore, the algorithm’s performance depends on the characteristics of the problem and parameter configurations. Therefore, rigorous experimentation and analysis are essential in practical applications to assess the positive impact of incorporating Gaussian Chaos on the DBO algorithm’s performance.

**Figure 1:**
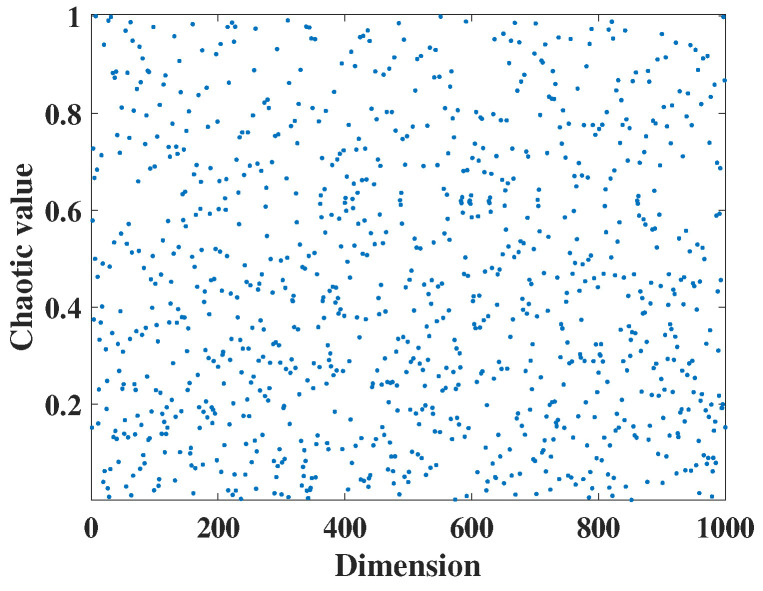
The Gaussian chaotic distribution plot.

In this study, the initial positions of dung beetles are initialized using Gaussian mapping. Initially, the obtained values are projected into the chaotic variable space using Gaussian mapping relations. Then, the generated chaotic values are mapped to the algorithm’s initial space through a nonlinear transformation. The Gaussian distribution plot is illustrated in Figure 1. The specific expression for Gaussian mapping is as follows:

(8) $$x_{i+1}=\left\{\begin{array}{c} 1,\;x_{i}=0\\ \frac{1}{\mathrm{mod}(x_{i},1)},\;\mathrm{otherwise} \end{array}\right.$$

### 3.3. The Spiral Search Strategy

Within the DBO algorithm, if dung beetles choose to reproduce their offspring within the current spawning region, it can lead to rapid population convergence within a short timeframe. However, this strategy also leads to a decrease in population diversity, increasing the risk of the algorithm falling into local optima. Therefore, improvements are necessary within the reproductive phase of the DBO algorithm.

Taking inspiration from the hunting behavior of whale populations within the Whale Optimization Algorithm, individual whales employ a spiral search strategy during the iterative process to update their positions relative to prey. This approach not only expedites the algorithm’s convergence but also enhances individual diversity. The specific formula for the whale-encircling-prey phase is as follows:

(9) $$\begin{array}{cc} \; & x_{i}\left(t+1\right)=D^{\prime }\cdot e^{cl}\cdot \cos\left(2\pi l\right)+X^{best1}\\ \; & D^{\prime }=\left|X^{best1}-x_{i}\left(t\right)\right| \end{array}$$

In the context of a logarithmic spiral, shape definition represents a constant and is a random number within the range of [−1, 1].

However, this strategy is highly susceptible to the influence of the parameter *c*. A larger *c* value can result in the rapid decay of the algorithm, leading to local optima, while a smaller *c* value can lead to a slow convergence of the algorithm. To address this issue, a parameter *r* for dynamic spiral search shape is introduced, and its definition is as follows:

(10) $$r=e^{c\cdot \cos(\frac{\pi t}{T_{\max}})}$$

The reproductive phase of the DBO algorithm has been enhanced by incorporating the spiral search strategy. The updated formula for the DBO algorithm is as follows:

(11) $$\begin{array}{cc} \; & x_{i}\left(t+1\right)=X^{best1}+e^{rl}\cdot \cos\left(2\pi l\right)\cdot b_{1}\times \left(x_{i}\left(t\right)-Lb^{*}\right)+e^{rl}\cdot \cos\left(2\pi l\right)\cdot b_{2}\times \left(x_{i}\left(t\right)-Ub^{*}\right) \end{array}$$

**Figure 2:**
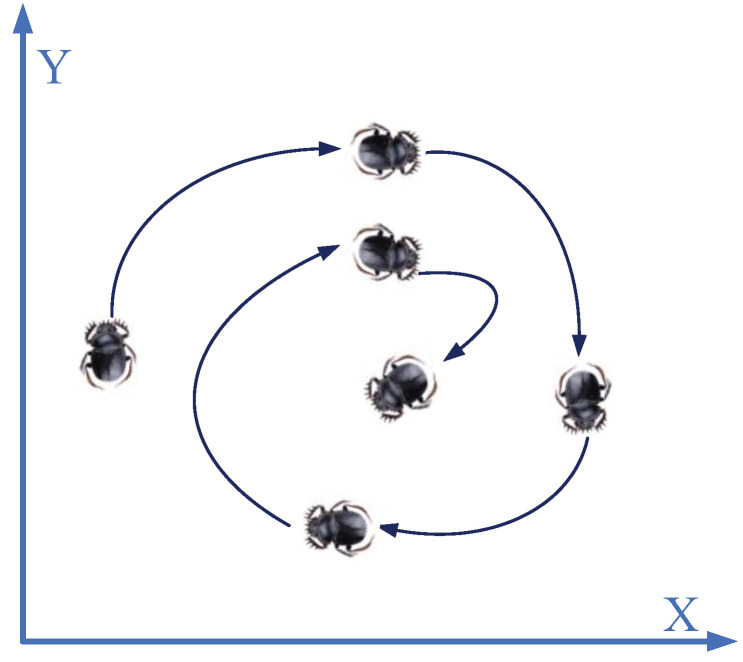
The dung beetle’s search trajectory.

Incorporating the dung beetle’s search path with a spiral pattern (as depicted in Figure 2) not only expedites the DBO algorithm’s convergence, but also augments the diversity of individuals.

### 3.4. Optimal Value-Guided Strategy

During the foraging phase of the DBO algorithm, the generation of candidate solutions is influenced by two random numbers. However, this results in an equal probability of generating both better and worse candidate solutions. To address this, we introduce the current best value to guide the generation of candidate solutions. The specific steps are as follows.

**Figure 3:**
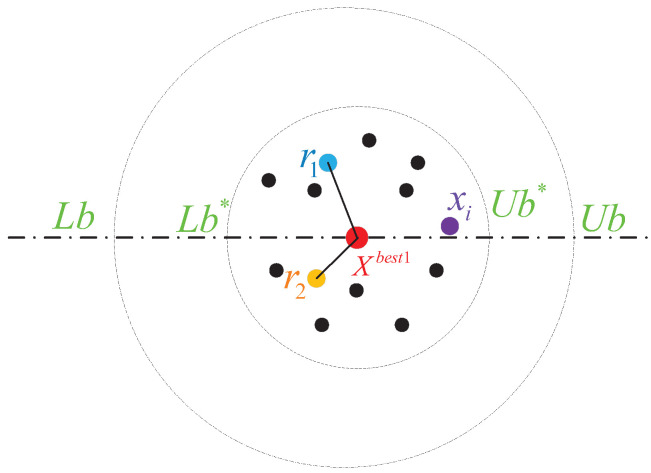
The dung beetle’s search trajectory.

Dung beetles forage as illustrated in Figure 3. During the foraging phase, dung beetles stochastically select two positions, denoted as $r_{1}$ and $r_{2}$, and subsequently explore the vicinity of these designated locations along with the position of the local optimum. This strategy considers both the local optimum and the randomly chosen individuals, thereby enhancing both search velocity and the quantity of search individuals. The revised formula for foraging dung beetles is as follows:

(12) $$\begin{array}{cc} \; & x_{i}\left(t+1\right)=x_{i}\left(t\right)+S_{1}\cdot C_{1}\times \left(x_{i}\left(t\right)-Lb^{b}\right)+S_{2}\cdot C_{2}\times \left(x_{i}\left(t\right)-Ub^{b}\right)+\lambda \times \left(X^{best1}-x_{i}\left(t\right)\right)\\ \; & S_{1}=e^{\frac{X^{best1}-x_{r_{1}}}{\left|X^{best1}\right|-\xi }}\\ \; & S_{2}=e^{\left(x_{r_{2}-}X^{best1}\right)} \end{array}$$

In this context, $x_{r_{1}}$ and $x_{r_{2}}$ represent the fitness values of $r_{1}$ and $r_{2}$, respectively. ξ stands for a small constant to prevent $\left(X^{best1}-x_{r_{1}}\right)/\left(\left|X^{best1}\right|-\xi \right)$ from being equal to zero. Additionally, it is important to note that $r_{1}$ is not equal to $r_{2}$, and λ=rand(1).

Incorporating this strategy demonstrates a unique characteristic as it combines information extracted from local optima with randomly selected individuals, resulting in a significant enhancement of both search speed and the number of search entities. More precisely, during the foraging phase, dung beetles randomly designate two positions, denoted as $r_{1}$ and $r_{2}$. They subsequently explore the nearby regions of these designated locations, in conjunction with the position of the local optimum. This approach expedites global exploration within the algorithm, allowing dung beetles to more effectively traverse the potential solution space.

### 3.5. Nonlinear Weighting

Although the DBO algorithm is relatively effective in addressing optimization problems, it is not immune to certain limitations. Firstly, DBO faces challenges in achieving a harmonious balance between its global exploration and local exploitation capabilities, making it prone to becoming entrapped in local optima. Secondly, its adaptability to various problem domains is suboptimal, displaying limited flexibility in dynamically adjusting search strategies. To overcome these drawbacks, introducing nonlinear weighting represents an effective strategy for improving the DBO algorithm. In the early stages, when the weight factor is relatively large, it enables the dung beetles to move closer to food sources, enhancing their exploration capabilities. As the search progresses, the weight factor decreases, leading the dung beetles to conduct more meticulous searches for food sources. This dynamic adjustment of the weight factor enables the DBO algorithm to strike a balance between global exploration and local exploitation, thereby enhancing its performance in tackling complex optimization problems. Following this improvement, the modified formula is as follows:

(13) $$\begin{array}{cc} \; & x_{i}\left(t+1\right)=X^{best2}+S\cdot g\cdot \left(\left|x_{i}\left(t\right)-X^{best1}\right|+\left|x_{i}\left(t\right)-X^{best2}\right|\right)\cdot \omega \\ \; & \omega =\omega_{\max}-\left(\omega_{\max}-\omega_{\min}\right)\cdot e^{\frac{1-t}{T_{\max}}} \end{array}$$

**Figure 4:**
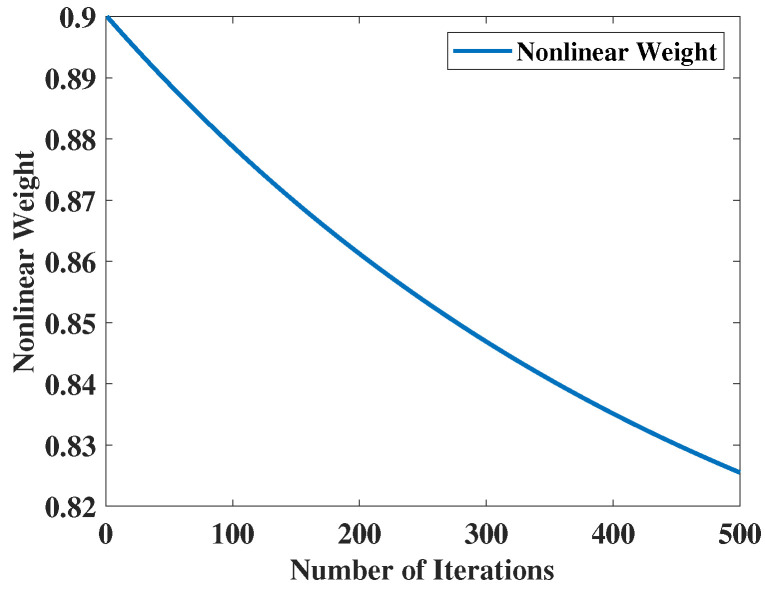
Nonlinear weight values.

In this context, $\omega_{\max}$ represents the maximum weighting factor, while $\omega_{\min}$ signifies the minimum weighting factor. In this study, the numerical value of $\omega_{\max}$ is 0.904, whereas $\omega_{\min}$ is assigned a numerical value of 0.782. Additionally, the simulation graph of the nonlinear weighting is depicted in Figure 4.

The introduction of nonlinear weights yields several notable enhancements to the DBO algorithm. Firstly, it confers heightened flexibility, enabling the algorithm to adaptively calibrate search strategies across different problem domains and at varying stages. As a result, the algorithm strikes a more harmonious balance between global exploration and local exploitation. Secondly, this incorporation of nonlinear weights results in performance improvements, including expedited convergence rates, augmented search capabilities, and enhanced solution quality. Most importantly, the utilization of nonlinear weights increases the DBO algorithm’s suitability for tackling complex problem landscapes, as it dynamically adjusts to variations in problem characteristics, ultimately bolstering the algorithm’s robustness.

### 3.6. ADBO Algorithm Implementation Steps

**Figure 5:**
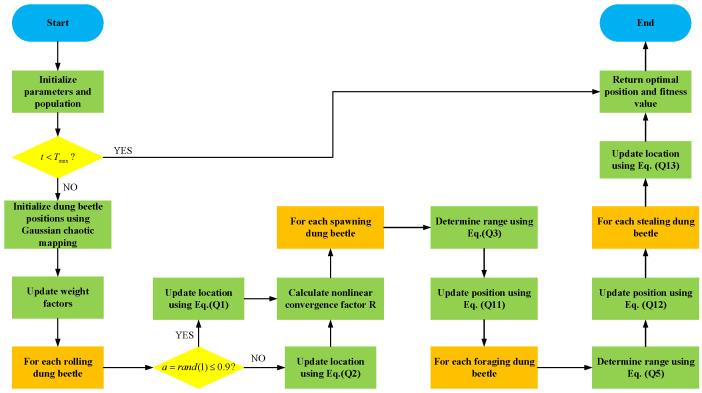
The ADBO algorithm.

The pseudocode for the ADBO algorithm with updated formulas is presented in Algorithm 2. The flowchart of the ADBO algorithm is shown in Figure 5.
**Algorithm 2** Framework of the ADBO Algorithm**Input:** Maximum iteration $T_{max}$, population size *N***Output:** Optimal position $X^{best2}$ and its corresponding fitness value $f_{\min}$1:Initialize the population of particles, indexed as i=1,2…N, and define relevant parameters, setting $\omega_{\max}$A and $\omega_{\min}$.2:**while** $t\leq T_{\max}$**do**3:   Initialize dung beetle positions using Gaussian chaotic mapping according to Equation (8).4:   Update the weight factors using Equation (13).5:   **for** *i* belonging to the rolling dung beetles group. **do**6:     a=rand(1)7:     **if** a≤0.9 **then**8:        Update the location of the rolling dung beetle using Equation (1).9:     **else**10:        Simulate rolling the ball in the presence of obstacles using Equation (2) to update the location.11:     **end if**12:   **end for**13:   Calculate the nonlinear convergence factor as $R=1-t/T_{max}$.14:   **for** *i* belonging to the spawning dung beetles group. **do**15:     Using Equation (3) to determine the range of spawning dung beetles and Equation (11) to update the position of the spawning dung beetles.16:   **end for**17:   **for** *i* belonging to the foraging dung beetles group. **do**18:     Determine the range of foraging dung beetles using Equation (5) and update the position of the foraging dung beetles using Equation (12).19:   **end for**20:   **for** *i* belonging to the stealing dung beetles group. **do**21:     Update the location of the stealing dung beetle using Equation (13).22:   **end for**23:**end while**24:**return** Return the optimal position $X^{best2}$ and its corresponding fitness value $f_{\min}$.

### 3.7. The Time Complexity of ADBO

The time complexity of the ADBO algorithm can be derived by analyzing its primary components. The initialization phase, which includes setting the population and defining parameters, has a complexity of O(N). The main loop runs for $T_{\max}$ iterations, where each iteration involves several key operations:

- Initializing dung beetle positions using Gaussian chaotic mapping: O(N).
- Updating the weight factors: O(1).
- Updating the rolling, spawning, foraging, and stealing dung beetles, each with a complexity of $O(N_{r})$, $O(N_{s})$, $O(N_{f})$, and $O(N_{t})$, respectively, where $N=N_{r}+N_{s}+N_{f}+N_{t}$.

Thus, the complexity of each iteration is O(N). Since the main loop runs for $T_{\max}$ iterations, the overall time complexity of the ADBO algorithm is $O(T_{\max}\cdot N)$.

## 4. Experimental Results and Discussion

In this study, we evaluate the performance of the ADBO algorithm through a comparative analysis with seven benchmark algorithms using a set of 29 test functions extracted from CEC2017 (for detailed function information, please refer to Table 1). These benchmark algorithms include ‘SSA’, ‘HHO’, ‘BOA’, ‘OMA’, ‘WOA’, ‘SCA’, and ‘DBO’. The parameter configurations for these benchmark algorithms are documented in Table 2. To ensure fair experimentation, we set the initial population size uniformly to 30 for all algorithms, with a fixed maximum iteration count of 500. To mitigate the influence of random variations, we employ assessment criteria that encompass both the mean and standard deviation of solution outcomes. These criteria are obtained by independently running each of the seven benchmark algorithms and the ADBO algorithm 100 times on each test function.

We utilized the MATLAB (R2022a) programming environment to execute the source code. For the BOA, the parameters are denoted as follows: signifies the power exponent, and indicates the perceptual mode. Regarding the DBO algorithm, the parameters are designated as follows: “RDB” represents the dung beetle population, “EDB” is assigned to the ovipositing dung beetle, “FDB” refers to the foraging dung beetle, and “SDB” represents the scavenging dung beetle. In Table 5 and Table 6, the metric rows depict the average solution rankings. Specifically, a ranking of 1 signifies that, following 500 iterations, the algorithm has achieved the finest mean solution value, highlighting its superior search capabilities, for dimensions Dim = 30 and Dim = 100, respectively.

### 4.1. Results and Analysis of Cec2017 Benchmark Functions

CEC2017 comprises a set of 29 single-objective benchmark functions. These functions cover a wide range of characteristics: F1 and F2 are unimodal functions, F3 to F9 are simple multimodal functions, F10 to F19 are hybrid functions, and F20 to F29 are composite functions. In Table 3 and Table 4, we present the mean rankings of solution outcomes for the ADBO algorithm and its comparative algorithms. These rankings are based on 100 independent runs for each function within CEC2017. A detailed analysis of the test results is presented below.

**Table 3:** CEC2017 test results: 30 dimensions. Bold text indicates the optimal values.

		Dim = 30							
		SSA	HHO	BOA	OMA	WOA	SCA	DBO	ADBO
F1	min	1.11E+02	1.50E+08	5.11E+10	3.31E+08	1.52E+06	1.38E+10	7.71E+04	4.06E+04
	mean	**4.75E+03**	4.23E+08	7.75E+10	2.22E+09	4.33E+08	2.05E+10	2.23E+08	6.37E+04
	std	2.74E+07	6.18E+16	7.23E+19	4.47E+18	6.31E+17	1.62E+19	1.65E+16	2.31E+14
	degree	1	4	8	6	5	7	3	2
F2	min	3.23E+04	3.49E+04	7.68E+04	3.98E+04	4.83E+03	6.20E+04	5.93E+04	3.42E+04
	mean	4.70E+04	5.74E+04	8.68E+05	6.41E+04	**1.28E+04**	8.75E+04	9.09E+04	6.30E+04
	std	3.74E+07	5.75E+07	1.09E+13	1.94E+08	2.48E+07	2.99E+08	3.49E+08	1.86E+08
	degree	2	3	8	5	1	6	7	4
F3	min	4.69E+02	5.82E+02	6.10E+03	5.87E+02	4.76E+02	1.71E+03	5.24E+02	4.23E+02
	mean	4.96E+02	7.36E+02	1.62E+04	7.93E+02	5.75E+02	2.70E+03	6.43E+02	**4.56E+02**
	std	4.58E+02	1.46E+04	3.60E+07	2.38E+04	2.59E+03	6.11E+05	5.46E+03	2.65E+03
	degree	2	5	8	6	3	7	4	1
F4	min	6.25E+02	7.21E+02	8.81E+02	6.28E+02	6.25E+02	7.78E+02	6.52E+02	5.95E+02
	mean	7.62E+02	7.82E+02	9.83E+02	7.06E+02	6.91E+02	8.31E+02	7.53E+02	**6.87E+02**
	std	2.68E+03	6.11E+02	1.98E+03	1.56E+03	1.00E+03	7.08E+02	1.75E+03	2.48E+03
	degree	5	6	8	3	2	7	4	1
F5	min	6.20E+02	6.52E+02	6.84E+02	6.16E+02	6.32E+02	6.55E+02	6.28E+02	6.25E+02
	mean	6.47E+02	6.69E+02	7.09E+02	6.30E+02	6.49E+02	6.63E+02	6.52E+02	**6.24E+02**
	std	1.95E+02	3.87E+01	2.08E+02	7.84E+01	6.57E+01	4.60E+01	1.18E+02	1.28E+02
	degree	3	7	8	2	4	6	5	1
F6	min	1.03E+03	1.16E+03	1.40E+03	1.00E+03	9.20E+02	1.13E+03	8.48E+02	9.17E+02
	mean	1.23E+03	1.32E+03	1.55E+03	1.19E+03	1.06E+03	1.25E+03	1.02E+03	**9.85E+02**
	std	9.94E+03	4.83E+03	5.86E+03	8.02E+03	6.85E+03	7.10E+03	6.00E+03	5.69E+03
	degree	5	7	8	4	3	6	2	1
F7	min	9.36E+02	9.62E+02	1.16E+03	9.36E+02	8.90E+02	1.06E+03	9.14E+02	8.80E+02
	mean	9.81E+02	9.89E+02	1.23E+03	9.81E+02	9.42E+02	1.10E+03	1.03E+03	**9.21E+02**
	std	1.07E+03	2.07E+02	1.53E+03	6.28E+02	4.25E+02	4.76E+02	2.57E+03	1.40E+03
	degree	3	5	8	4	2	7	6	1
F8	min	3.85E+03	6.65E+03	1.31E+04	1.87E+03	1.93E+03	5.11E+03	2.94E+03	2.40E+03
	mean	5.27E+03	8.51E+03	1.71E+04	3.83E+03	4.00E+03	8.65E+03	7.23E+03	**3.53E+03**
	std	1.54E+05	1.18E+06	3.30E+06	1.27E+06	7.36E+05	3.29E+06	6.98E+06	2.27E+06
	degree	4	6	8	2	3	7	5	1
F9	min	3.57E+03	4.88E+03	9.22E+03	6.72E+03	3.67E+03	8.15E+03	4.57E+03	3.65E+03
	mean	5.39E+03	6.36E+03	1.03E+04	8.35E+03	5.24E+03	8.81E+03	6.67E+03	**4.18E+03**
	std	6.63E+05	4.39E+05	1.86E+05	4.01E+05	9.30E+05	1.39E+05	1.40E+06	1.07E+06
	degree	3	4	8	6	2	7	5	1
F10	min	1.16E+03	1.32E+03	5.63E+03	1.21E+03	1.16E+03	2.27E+03	1.31E+03	1.14E+03
	mean	1.29E+03	1.63E+03	2.63E+04	1.37E+03	**1.27E+03**	3.68E+03	1.91E+03	1.35E+03
	std	5.28E+03	5.31E+04	1.65E+08	7.55E+03	6.75E+03	1.03E+06	2.90E+05	6.55E+03
	degree	2	5	8	4	1	7	6	3
F11	min	3.06E+04	1.12E+07	1.08E+10	2.00E+06	2.17E+05	1.52E+09	1.65E+06	1.42E+05
	mean	**1.21E+06**	8.91E+07	2.05E+10	2.08E+07	2.04E+06	2.84E+09	5.65E+07	8.71E+06
	std	7.83E+11	5.84E+15	2.91E+19	4.16E+14	3.92E+12	5.68E+17	6.26E+15	1.44E+14
	degree	1	6	8	4	2	7	5	3
F12	min	3.61E+03	4.75E+05	4.33E+09	1.15E+04	5.20E+03	5.71E+08	2.46E+04	7.43E+03
	mean	3.22E+04	1.37E+06	1.89E+10	2.33E+05	**1.82E+04**	1.07E+09	5.03E+06	1.95E+05
	std	8.01E+08	1.33E+12	5.57E+19	5.38E+11	2.62E+08	9.26E+16	1.03E+14	3.83E+11
	degree	2	5	8	4	1	7	6	3
F13	min	9.77E+03	3.87E+04	2.45E+06	2.56E+03	1.61E+03	8.34E+04	6.42E+03	2.29E+03
	mean	6.49E+04	1.45E+06	2.21E+07	3.62E+04	**5.06E+03**	8.72E+05	4.70E+05	2.55E+05
	std	1.61E+09	1.57E+12	2.23E+14	1.81E+09	4.70E+07	4.06E+11	1.13E+12	5.99E+10
	degree	3	7	8	2	1	6	5	4
F14	min	2.15E+03	3.63E+04	5.21E+08	2.22E+03	1.87E+03	2.02E+06	1.07E+04	1.96E+03
	mean	1.29E+04	1.26E+05	3.21E+09	9.53E+03	**4.18E+03**	6.64E+07	1.17E+05	1.24E+04
	std	1.25E+08	4.63E+09	2.82E+18	8.79E+07	6.84E+06	2.37E+15	4.67E+10	1.35E+08
	degree	4	6	8	2	1	7	5	3
F15	min	2.20E+03	2.78E+03	4.11E+03	2.74E+03	2.16E+03	3.52E+03	2.25E+03	1.80E+03
	mean	2.94E+03	3.59E+03	5.51E+03	3.40E+03	2.75E+03	4.20E+03	3.27E+03	**2.61E+03**
	std	6.92E+04	2.34E+05	6.02E+05	9.44E+04	9.39E+04	8.97E+04	1.75E+05	6.74E+04
	degree	3	6	8	5	2	7	4	1
F16	min	1.87E+03	2.17E+03	2.93E+03	1.97E+03	1.91E+03	2.09E+03	2.12E+03	1.70E+03
	mean	2.37E+03	2.69E+03	3.88E+03	**2.24E+03**	2.43E+03	2.82E+03	2.62E+03	2.57E+03
	std	6.54E+04	1.06E+05	2.22E+05	2.64E+04	8.66E+04	6.99E+04	5.54E+04	9.51E+04
	degree	2	6	8	1	3	7	5	4
F17	min	5.11E+04	8.62E+04	5.57E+07	7.25E+04	1.43E+04	2.74E+06	1.39E+05	2.30E+04
	mean	7.09E+05	4.78E+06	4.24E+08	4.14E+05	**1.13E+05**	1.18E+07	3.27E+06	1.22E+06
	std	8.66E+11	3.90E+13	8.91E+16	2.48E+11	1.38E+10	4.65E+13	6.13E+13	4.21E+12
	degree	3	6	8	2	1	7	5	4
F18	min	2.05E+03	1.10E+05	9.23E+08	2.73E+03	2.06E+03	2.61E+07	4.77E+03	2.08E+03
	mean	1.42E+04	1.68E+06	3.62E+09	1.57E+04	**7.17E+03**	8.52E+07	1.78E+07	1.21E+04
	std	2.02E+08	2.31E+12	3.37E+18	1.28E+08	2.80E+07	2.24E+15	4.33E+15	1.40E+08
	degree	3	5	8	4	1	7	6	2
F19	min	2.47E+03	2.42E+03	3.03E+03	2.43E+03	2.27E+03	2.55E+03	2.49E+03	2.03E+03
	mean	2.75E+03	2.89E+03	3.57E+03	2.64E+03	2.52E+03	2.95E+03	2.81E+03	**2.41E+03**
	std	3.84E+04	6.51E+04	4.86E+04	1.31E+04	2.05E+04	2.48E+04	2.43E+04	5.07E+04
	degree	4	6	8	3	2	7	5	1
F20	min	2.42E+03	2.46E+03	2.59E+03	2.41E+03	2.38E+03	2.57E+03	2.35E+03	2.19E+03
	mean	2.51E+03	2.58E+03	2.77E+03	2.49E+03	2.47E+03	2.62E+03	2.55E+03	**2.47E+03**
	std	2.55E+03	2.75E+03	4.45E+03	1.01E+03	2.08E+03	7.59E+02	4.28E+03	4.30E+03
	degree	4	6	8	3	2	7	5	1
F21	min	2.30E+03	2.75E+03	6.67E+03	2.46E+03	2.32E+03	3.94E+03	2.39E+03	2.22E+03
	mean	5.50E+03	7.68E+03	1.11E+04	**2.76E+03**	2.96E+03	9.92E+03	5.00E+03	2.98E+03
	std	4.89E+06	1.19E+06	1.35E+06	4.87E+04	1.48E+06	2.41E+06	5.51E+06	2.27E+06
	degree	5	6	8	1	2	7	4	3
F22	min	2.76E+03	3.03E+03	3.09E+03	2.82E+03	2.82E+03	2.99E+03	2.82E+03	2.54E+03
	mean	2.91E+03	3.30E+03	3.42E+03	**2.90E+03**	2.95E+03	3.07E+03	3.03E+03	2.97E+03
	std	8.61E+03	2.38E+04	2.36E+04	1.83E+03	6.68E+03	2.37E+03	6.73E+03	5.91E+03
	degree	2	7	8	1	3	6	5	4
F23	min	2.92E+03	3.27E+03	3.25E+03	2.99E+03	2.93E+03	3.17E+03	3.00E+03	2.82E+03
	mean	3.05E+03	3.52E+03	3.50E+03	3.10E+03	3.11E+03	3.26E+03	3.18E+03	**2.79E+03**
	std	6.07E+03	2.42E+04	3.39E+04	2.86E+03	7.32E+03	1.24E+03	1.06E+04	9.22E+03
	degree	2	8	7	3	4	6	5	1
F24	min	2.88E+03	2.95E+03	4.23E+03	3.00E+03	2.90E+03	3.29E+03	2.89E+03	2.61E+03
	mean	**2.90E+03**	3.01E+03	5.69E+03	3.08E+03	2.96E+03	3.54E+03	2.99E+03	2.96E+03
	std	1.95E+02	1.01E+03	6.06E+05	3.48E+03	1.23E+03	3.82E+04	4.49E+03	1.14E+03
	degree	1	5	8	6	2	7	4	3
F25	min	5.12E+03	3.92E+03	9.34E+03	5.43E+03	3.37E+03	7.22E+03	5.38E+03	3.38E+03
	mean	6.46E+03	7.93E+03	1.15E+04	6.38E+03	6.59E+03	7.92E+03	7.12E+03	**6.31E+03**
	std	5.91E+05	1.80E+06	1.10E+06	2.09E+05	1.84E+06	2.84E+05	4.68E+05	1.16E+06
	degree	3	7	8	2	4	6	5	1
F26	min	3.22E+03	3.28E+03	3.57E+03	3.28E+03	3.23E+03	3.41E+03	3.25E+03	3.10E+03
	mean	**3.26E+03**	3.63E+03	4.08E+03	3.34E+03	3.36E+03	3.56E+03	3.32E+03	3.33E+03
	std	1.57E+03	3.74E+04	1.38E+05	1.43E+03	8.02E+03	9.12E+03	3.28E+03	6.02E+03
	degree	1	7	8	4	5	6	2	3
F27	min	3.20E+03	3.32E+03	5.79E+03	3.38E+03	3.24E+03	3.94E+03	3.29E+03	3.29E+03
	mean	**3.23E+03**	3.49E+03	7.21E+03	3.52E+03	3.34E+03	4.48E+03	3.51E+03	3.37E+03
	std	5.08E+02	1.12E+04	6.92E+05	1.22E+04	2.27E+03	8.16E+04	1.47E+05	2.81E+03
	degree	1	4	8	6	2	7	5	3
F28	min	3.45E+03	4.37E+03	5.52E+03	3.84E+03	3.96E+03	4.50E+03	3.82E+03	3.49E+03
	mean	**4.15E+03**	5.02E+03	7.39E+03	4.23E+03	4.45E+03	5.15E+03	4.44E+03	4.43E+03
	std	1.31E+05	2.08E+05	9.72E+05	3.79E+04	5.99E+04	1.12E+05	1.46E+05	2.09E+05
	degree	1	6	8	2	5	7	4	3
F29	min	5.67E+03	6.28E+05	8.11E+08	3.66E+04	6.02E+03	8.47E+07	2.19E+04	1.99E+04
	mean	**1.73E+04**	1.32E+07	2.74E+09	2.67E+05	3.91E+04	1.97E+08	3.85E+06	2.77E+05
	std	9.47E+07	2.24E+14	2.34E+18	5.95E+10	2.98E+09	5.65E+15	1.83E+13	5.11E+11
	degree	1	6	8	3	2	7	5	4

**Table 4:** CEC2017 test results: 30 dimensions. Bold text indicates the optimal values.

		Dim = 30							
		SSA	HHO	BOA	OMA	WOA	SCA	DBO	ADBO
F1	min	1.83E+08	3.14E+10	2.75E+11	7.68E+10	2.70E+10	1.87E+11	2.24E+10	1.33E+10
	mean	**3.83E+08**	4.95E+10	2.95E+11	1.18E+11	6.48E+10	2.17E+11	7.86E+10	3.76E+10
	std	1.07E+16	6.42E+19	2.20E+19	4.76E+20	1.95E+20	2.38E+20	4.78E+21	1.34E+20
	degree	1	3	8	6	4	7	5	2
F2	min	3.48E+05	3.15E+05	8.62E+05	3.56E+05	1.75E+05	4.74E+05	3.56E+05	3.27E+05
	mean	7.35E+05	3.61E+05	1.88E+10	4.17E+05	**2.43E+05**	6.01E+05	7.75E+05	4.00E+05
	std	1.43E+10	6.12E+09	3.30E+21	1.13E+09	4.18E+08	5.73E+09	9.76E+10	1.24E+10
	degree	6	2	8	4	1	5	7	3
F3	min	8.59E+02	6.66E+03	9.04E+04	1.06E+04	2.01E+03	3.79E+04	3.75E+03	1.01E+03
	mean	**1.01E+03**	8.99E+03	1.18E+05	1.74E+04	7.45E+03	5.11E+04	1.56E+04	2.97E+03
	std	9.17E+03	1.62E+06	1.59E+08	1.76E+07	9.11E+06	4.62E+07	3.00E+08	2.87E+06
	degree	1	4	8	6	3	7	5	2
F4	min	1.29E+03	1.56E+03	2.20E+03	1.46E+03	1.33E+03	1.97E+03	1.19E+03	1.14E+03
	mean	1.37E+03	1.67E+03	2.29E+03	1.72E+03	1.46E+03	2.07E+03	1.70E+03	**1.31E+03**
	std	1.78E+03	2.69E+03	3.29E+03	1.32E+04	3.95E+03	3.14E+03	5.44E+04	5.53E+03
	degree	2	4	8	6	3	7	5	1
F5	min	6.62E+02	6.85E+02	7.09E+02	6.72E+02	6.64E+02	6.95E+02	6.62E+02	6.07E+02
	mean	6.66E+02	6.91E+02	7.27E+02	6.87E+02	6.71E+02	7.02E+02	6.77E+02	**6.51E+02**
	std	7.00E+00	1.84E+01	7.51E+01	6.23E+01	1.14E+01	1.07E+01	1.15E+02	3.18E+01
	degree	2	6	8	5	3	7	4	1
F6	min	2.58E+03	3.46E+03	4.05E+03	3.33E+03	2.96E+03	3.57E+03	2.55E+03	2.73E+03
	mean	3.19E+03	3.75E+03	4.27E+03	4.25E+03	3.20E+03	4.05E+03	**2.98E+03**	3.14E+03
	std	2.80E+04	1.67E+04	6.47E+03	1.82E+05	1.35E+04	4.95E+04	4.38E+04	3.08E+04
	degree	3	5	8	7	4	6	1	2
F7	min	1.64E+03	2.00E+03	2.62E+03	1.81E+03	1.77E+03	2.29E+03	1.75E+03	1.69E+03
	mean	1.84E+03	2.13E+03	2.77E+03	2.02E+03	1.89E+03	2.43E+03	2.10E+03	**1.79E+03**
	std	2.58E+03	3.14E+03	7.42E+03	1.65E+04	5.02E+03	4.60E+03	6.04E+04	1.47E+04
	degree	2	6	8	4	3	7	5	1
F8	min	2.42E+04	6.06E+04	8.96E+04	6.11E+04	2.59E+04	6.99E+04	4.75E+04	3.95E+04
	mean	**2.53E+04**	6.95E+04	1.08E+05	7.36E+04	3.04E+04	8.98E+04	7.60E+04	4.96E+04
	std	3.99E+05	2.19E+07	7.46E+07	7.56E+07	8.36E+06	9.74E+07	1.03E+08	1.45E+08
	degree	1	4	8	5	2	7	6	3
F9	min	1.40E+04	2.05E+04	3.33E+04	2.82E+04	1.75E+04	3.09E+04	1.94E+04	1.70E+04
	mean	**1.73E+04**	2.47E+04	3.51E+04	3.21E+04	1.94E+04	3.31E+04	2.84E+04	1.79E+04
	std	1.25E+06	5.18E+06	9.98E+05	8.34E+05	1.52E+06	4.17E+05	2.36E+07	1.58E+07
	degree	1	4	8	6	3	7	5	2
F10	min	3.08E+04	7.34E+04	6.10E+05	6.63E+04	1.26E+04	1.34E+05	1.40E+05	4.26E+04
	mean	7.62E+04	1.41E+05	9.69E+06	1.03E+05	**3.57E+04**	1.85E+05	2.30E+05	5.47E+04
	std	5.12E+08	1.10E+09	1.40E+15	2.89E+08	9.99E+07	9.62E+08	3.30E+09	8.87E+08
	degree	3	5	8	4	1	6	7	2
F11	min	7.17E+07	5.31E+09	2.11E+11	1.01E+10	1.19E+09	7.93E+10	2.95E+09	2.48E+08
	mean	**1.88E+08**	1.14E+10	2.51E+11	2.05E+10	8.44E+09	1.03E+11	7.34E+09	2.64E+09
	std	4.75E+15	2.29E+19	2.12E+20	3.51E+19	4.61E+19	1.32E+20	4.52E+18	1.02E+19
	degree	1	5	8	6	4	7	3	2
F12	min	2.24E+04	6.36E+07	5.08E+10	3.47E+08	4.79E+05	9.56E+09	1.81E+07	7.67E+04
	mean	**6.46E+04**	2.32E+08	6.25E+10	1.21E+09	1.52E+08	1.69E+10	3.22E+08	2.89E+06
	std	9.55E+09	3.62E+16	1.52E+19	9.75E+17	7.87E+16	1.83E+19	4.87E+16	3.46E+13
	degree	1	4	8	6	3	7	5	2
F13	min	8.28E+05	4.89E+06	1.09E+08	1.18E+06	5.08E+05	1.76E+07	2.85E+06	1.01E+06
	mean	2.10E+06	1.12E+07	4.40E+08	3.96E+06	**1.72E+06**	6.25E+07	2.00E+07	2.05E+06
	std	7.27E+11	1.19E+13	5.79E+16	4.22E+12	8.34E+11	8.33E+14	1.68E+14	1.39E+13
	degree	3	5	8	4	1	7	6	2
F14	min	9.59E+03	6.90E+06	1.99E+10	6.79E+06	1.22E+04	3.10E+09	1.81E+05	2.64E+04
	mean	**2.21E+04**	2.13E+07	3.56E+10	4.96E+07	6.13E+06	5.94E+09	1.02E+08	3.88E+05
	std	1.44E+08	6.13E+14	3.00E+19	2.08E+15	4.57E+14	2.93E+18	2.48E+16	1.05E+12
	degree	1	4	8	5	3	7	6	2
F15	min	4.61E+03	7.96E+03	1.61E+04	7.59E+03	5.48E+03	1.30E+04	7.13E+03	4.05E+03
	mean	6.17E+03	1.03E+04	2.39E+04	9.97E+03	7.40E+03	1.48E+04	9.34E+03	**5.75E+03**
	std	6.19E+05	1.42E+06	9.70E+06	1.57E+06	1.09E+06	6.51E+05	1.82E+06	1.14E+06
	degree	2	6	8	5	3	7	4	1
F16	min	4.82E+03	6.14E+03	3.10E+06	4.64E+03	5.02E+03	1.41E+04	7.59E+03	3.02E+03
	mean	5.98E+03	9.17E+03	3.43E+07	6.61E+03	6.80E+03	9.06E+04	9.58E+03	**5.74E+03**
	std	4.71E+05	6.12E+07	1.25E+15	7.55E+05	1.18E+06	9.13E+09	4.03E+06	1.09E+06
	degree	2	5	8	3	4	7	6	1
F17	min	3.81E+05	2.54E+06	1.92E+08	1.05E+06	1.05E+06	4.79E+07	3.84E+06	1.58E+06
	mean	2.68E+06	9.22E+06	7.52E+08	6.05E+06	2.94E+06	1.34E+08	1.97E+07	**1.79E+06**
	std	1.42E+12	1.84E+13	1.27E+17	1.23E+13	3.39E+12	2.90E+15	1.11E+14	1.24E+13
	degree	2	5	8	4	3	7	6	1
F18	min	2.88E+03	1.33E+07	2.44E+10	4.57E+06	7.51E+04	3.14E+09	1.43E+07	4.15E+04
	mean	**2.87E+04**	3.92E+07	3.44E+10	4.83E+07	1.26E+07	5.30E+09	1.48E+08	1.46E+06
	std	5.28E+09	3.32E+14	2.75E+19	1.31E+15	2.93E+15	1.81E+18	1.43E+16	1.33E+12
	degree	1	4	8	5	3	7	6	2
F19	min	4.58E+03	4.92E+03	8.72E+03	6.65E+03	4.16E+03	7.41E+03	5.45E+03	4.02E+03
	mean	6.06E+03	6.12E+03	9.26E+03	7.45E+03	5.25E+03	8.14E+03	7.28E+03	**5.25E+03**
	std	3.60E+05	2.69E+05	6.94E+04	1.10E+05	3.39E+05	1.11E+05	6.12E+05	5.82E+05
	degree	3	4	8	6	2	7	5	1
F20	min	3.35E+03	4.03E+03	4.46E+03	3.18E+03	3.33E+03	3.99E+03	3.73E+03	3.03E+03
	mean	3.66E+03	4.40E+03	4.96E+03	3.45E+03	3.58E+03	4.21E+03	4.04E+03	**3.39E+03**
	std	4.69E+04	3.34E+04	4.69E+04	2.00E+04	2.36E+04	1.18E+04	3.73E+04	4.87E+04
	degree	4	7	8	2	3	6	5	1
F21	min	1.40E+04	2.45E+04	3.54E+04	3.31E+04	1.85E+04	3.38E+04	2.07E+04	2.01E+04
	mean	**1.94E+04**	2.78E+04	3.76E+04	3.48E+04	2.35E+04	3.53E+04	2.82E+04	2.73E+04
	std	2.89E+06	2.01E+06	1.30E+06	4.72E+05	2.94E+06	6.16E+05	2.46E+07	9.05E+06
	degree	1	4	8	6	2	7	5	3
F22	min	3.75E+03	5.43E+03	5.55E+03	3.93E+03	3.98E+03	5.05E+03	4.49E+03	3.75E+03
	mean	4.24E+03	5.95E+03	6.53E+03	4.20E+03	4.59E+03	5.26E+03	4.81E+03	**4.17E+03**
	std	3.78E+04	1.33E+05	4.27E+05	2.43E+04	8.34E+04	1.69E+04	3.07E+04	1.15E+05
	degree	3	7	8	2	4	6	5	1
F23	min	4.56E+03	7.05E+03	7.35E+03	5.23E+03	5.43E+03	6.93E+03	5.28E+03	4.91E+03
	mean	5.23E+03	8.57E+03	9.45E+03	5.92E+03	5.98E+03	7.43E+03	6.30E+03	**5.19E+03**
	std	1.26E+05	2.61E+05	3.70E+06	1.86E+05	9.61E+04	8.67E+04	2.40E+05	2.54E+05
	degree	2	7	8	3	4	6	5	1
F24	min	3.54E+03	5.91E+03	2.47E+04	9.14E+03	5.46E+03	1.82E+04	5.20E+03	4.73E+03
	mean	**3.69E+03**	6.78E+03	3.22E+04	1.23E+04	7.67E+03	2.28E+04	1.15E+04	5.72E+03
	std	6.90E+03	2.15E+05	9.35E+06	5.86E+06	1.12E+06	5.41E+06	5.86E+07	7.73E+05
	degree	1	3	8	6	4	7	5	2
F25	min	4.86E+03	2.83E+04	5.06E+04	2.95E+04	2.64E+04	3.46E+04	1.97E+04	2.01E+04
	mean	**2.10E+04**	3.23E+04	5.81E+04	3.65E+04	3.18E+04	4.16E+04	2.63E+04	2.61E+04
	std	5.17E+07	6.15E+06	1.33E+07	1.43E+07	6.10E+06	8.64E+06	1.29E+07	1.64E+07
	degree	1	5	8	6	4	7	3	2
F26	min	3.62E+03	5.85E+03	8.14E+03	4.71E+03	4.34E+03	7.44E+03	4.02E+03	3.70E+03
	mean	**3.89E+03**	7.65E+03	1.16E+04	5.47E+03	5.41E+03	8.70E+03	4.63E+03	4.59E+03
	std	6.25E+04	2.59E+06	2.17E+06	2.16E+05	3.44E+05	4.26E+05	2.51E+05	2.80E+05
	degree	1	6	8	5	4	7	3	2
F27	min	3.65E+03	7.61E+03	3.03E+04	1.09E+04	6.81E+03	2.19E+04	7.03E+03	4.87E+03
	mean	**3.80E+03**	9.27E+03	3.70E+04	1.47E+04	9.63E+03	2.66E+04	1.93E+04	7.56E+03
	std	9.55E+03	7.57E+05	6.98E+06	3.79E+06	2.39E+06	4.12E+06	3.80E+07	2.37E+06
	degree	1	3	8	5	4	7	6	2
F28	min	6.75E+03	1.04E+04	2.25E+05	9.23E+03	8.58E+03	2.14E+04	9.10E+03	5.66E+03
	mean	**7.73E+03**	1.31E+04	1.41E+06	1.27E+04	1.05E+04	4.15E+04	1.23E+04	9.81E+03
	std	2.41E+05	2.52E+06	1.07E+12	3.18E+06	8.00E+05	6.38E+08	2.55E+06	8.13E+05
	degree	1	6	8	5	3	7	4	2
F29	min	1.89E+05	3.34E+08	4.47E+10	3.43E+08	8.25E+06	6.19E+09	6.66E+07	5.10E+06
	mean	**6.18E+05**	7.95E+08	5.66E+10	2.09E+09	1.36E+08	1.33E+10	2.66E+08	7.45E+07
	std	1.28E+11	1.03E+17	2.23E+19	2.76E+18	3.09E+16	9.88E+18	1.95E+16	6.57E+16
	degree	1	5	8	6	3	7	4	2

### 4.2. Analysis of Statistical Results for Cec2017

Similarly, the experimental results for both the 30- and 100-dimensional cases are presented in Table 3 and Table 4. These tables offer insights into the mean and standard deviation of objective function values for each respective algorithm. The findings from these experimental analyses are discussed in detail below:

1. In the 30-dimensional and 100-dimensional tests, ADBO’s performance on F1 and F2 is just slightly below that of SSA, but it significantly outperforms DBO.
2. In the 30-dimensional tests, the ADBO algorithm consistently secures top rankings in both mean and minimum values among the F3–F9 functions. However, in the 100-dimensional tests, ADBO’s performance slightly falls behind the SSA in functions F3, F6, and F9. Nevertheless, it is worth noting that ADBO continues to outperform the SSA in other functions. This underscores the exceptional performance of the ADBO algorithm, particularly in lower dimensions, where it consistently leads in both mean and minimum values, showcasing its robust capabilities for global search and optimization. These findings further emphasize the competitive edge and adaptability of the ADBO algorithm, solidifying its position as a versatile and powerful tool across diverse problem domains and complexities.
3. Similarly, ADBO excels in addressing mixed problems, as evidenced by its performance. Specifically, in 30-dimensional experiments focused on test functions F15, F19, and F20, ADBO establishes a significant lead. Additionally, across various other test functions, ADBO’s performance is comparable to that of SSA, showcasing its impressive competitiveness. Even when confronted with more challenging 100-dimensional experiments, ADBO consistently upholds its outstanding performance. Notably, in test functions F15 through F17, F19, and F20, it outperforms other optimization algorithms by a substantial margin. These results underscore the substantial competitive advantage of the ADBO algorithm in tackling high-dimensional problems, making it a valuable solution for practical engineering challenges such as drone and robot path planning.
4. Similarly, when tackling composite problems, ADBO proves its formidable competitiveness in experimental results involving functions F21–F29. In all 30-dimensional experiments, the ADBO algorithm consistently delivers competent performance. However, it truly distinguishes itself in the 100-dimensional experiments. Across all remaining functions, it outperforms other comparative algorithms, demonstrating exceptional capabilities. The sole exception to this pattern is in comparison to the SSA, where ADBO falls slightly short. These findings unequivocally underline the ADBO algorithm’s unique strengths and adaptability in addressing intricate composite problems. ADBO’s performance highlights its ability to efficiently navigate and optimize complex search spaces, positioning it as a promising solution for real-world challenges spanning diverse domains, from engineering to data analysis and beyond. Its capacity to excel in both 30-dimensional and 100-dimensional experiments underscores the algorithm’s versatility and potential to address a wide spectrum of complex optimization problems.

### 4.3. Comparison of Convergence Curves for Cec2017 Benchmark Functions

**Figure 6:**
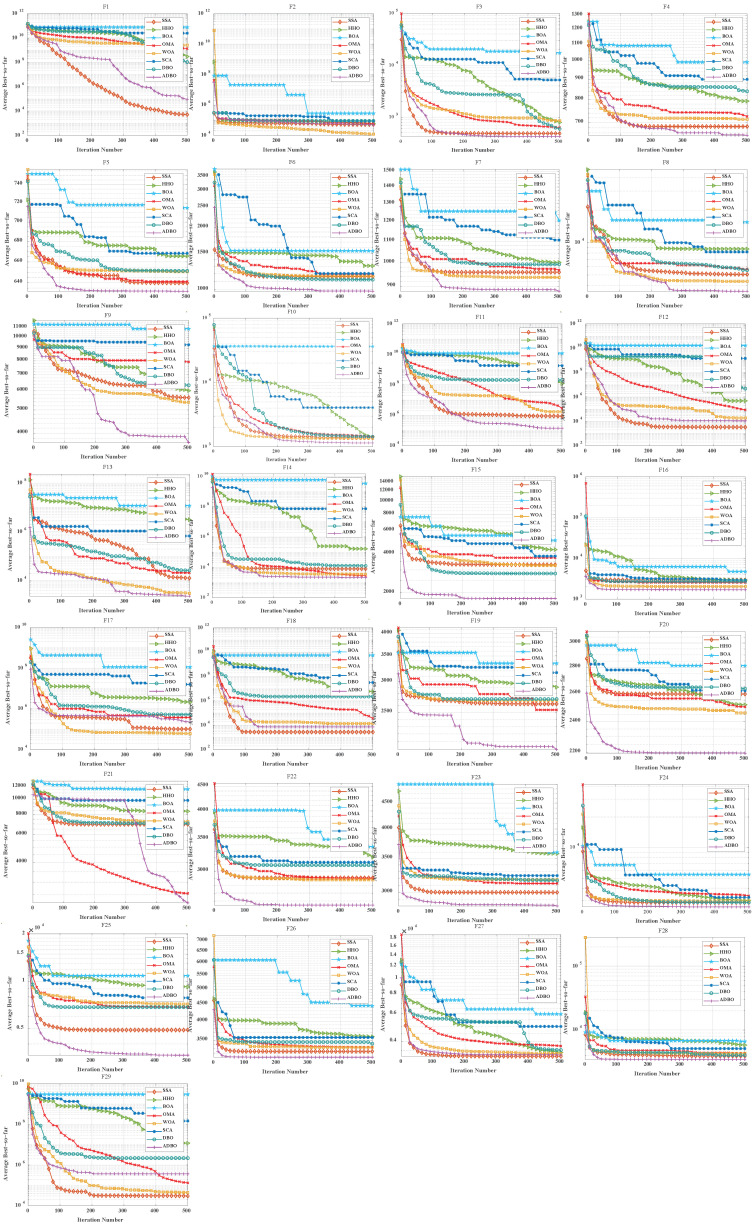
CEC2017 test curve chart (Dim = 30).

**Figure 7:**
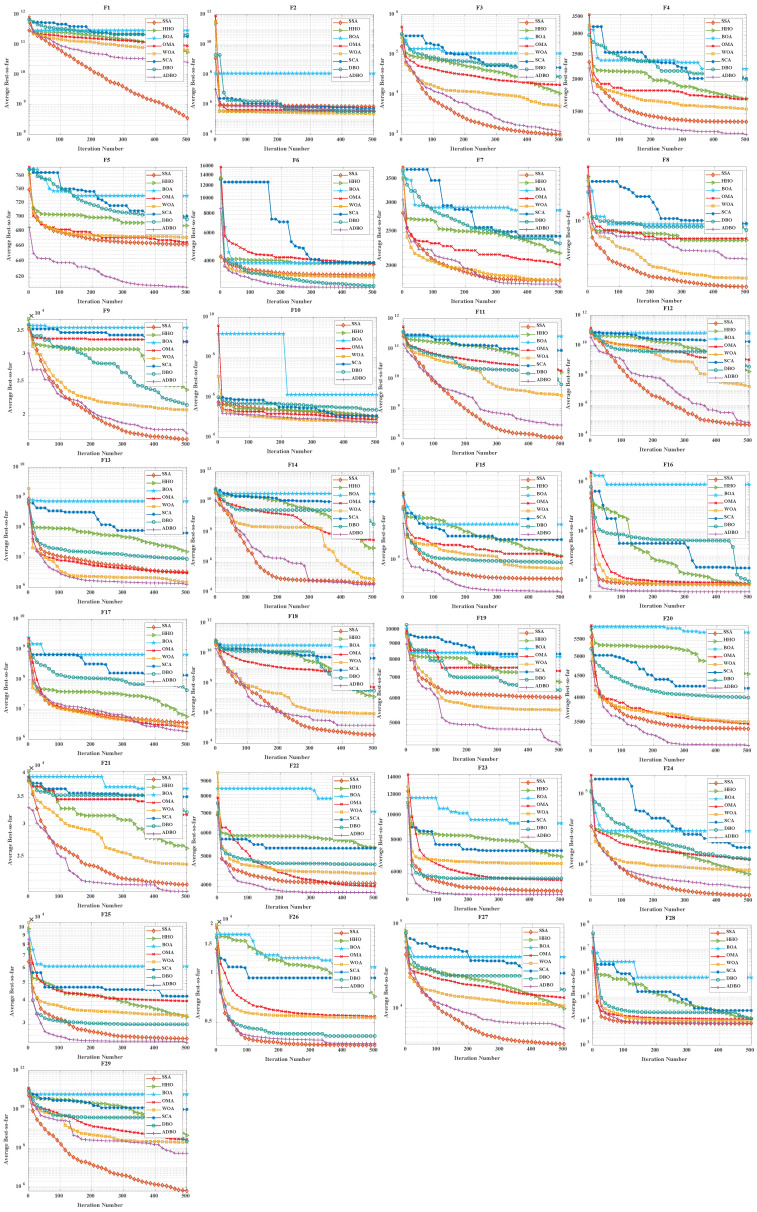
CEC2017 test curve chart (Dim = 100).

Similarly, Figure 6 (Dim = 30) and Figure 7 (Dim = 100) depict the convergence speed and accuracy of ADBO, SSA, HHO, BOA, WOA, SCA, and DBO in CEC2017. These figures clearly illustrate that ADBO achieves faster convergence, less fluctuation, and greater stability compared to other algorithms. This indicates that ADBO can quickly approach the optimal solution, enhancing its problem-solving efficiency and overall robustness. In most test scenarios, ADBO exhibits an accelerating convergence trend, suggesting that its search capability improves as iterations progress, enabling it to find better solutions more rapidly.

For 30-dimensional hybrid test functions, SSA’s search speed increases in the later stages, leading to slower convergence on functions F3 to F9 and F19 to F25. ADBO overtakes SSA promptly. In the case of 100-dimensional hybrid test functions, ADBO surpasses SSA only in functions F4 to F6, F15, F16, and F19 to F23.

For functions like F1, F2, and F29, WOA shows faster mid-period convergence due to reduced population diversity, but ADBO outperforms it after more than 2000 iterations. This difference may be attributed to the reduced spawning area, causing ADBO to perform less effectively in functions such as F2, F19, and others. The specific analysis is as follows:

1. The experimental results for the single-peaked problem F1 highlight ADBO’s strong performance in locating the global optimum and its efficiency. In the 30-dimensional experiments, ADBO slightly trails behind SSA on F1 but outperforms DBO by a significant margin. Notably, in the 100-dimensional experiments, ADBO exhibits exceptional performance on the F1 function, indicating its enhanced capability to discover and converge toward the global optimum in the context of single-peaked problems. This affirms its robust global exploration and exploitation potential. In the case of F2, while differences among the comparative algorithms are less pronounced, ADBO still demonstrates superiority over several other approaches.
2. The experiment results clearly demonstrate that the ADBO algorithm excels in solving straightforward multimodal problems (F3 to F10). In the 30-dimensional trials, ADBO shows a slight delay compared to other methods in functions F3, F4, F7, F8, F9, and F10. Nevertheless, it eventually overtakes SSA and WOA by rapidly discovering the best solution. ADBO consistently maintains a leading position in functions F5 and F6, swiftly reaching the optimum and securing the top rank. In the 100-dimensional experiments, ADBO continues to outperform other methods in functions F4, F5, and F6, albeit with a minor lag behind SSA in other functions. These outcomes further confirm the outstanding performance and resilience of the ADBO algorithm in tackling complex problems. Its robust abilities for global exploration and exploitation establish it as a formidable tool for addressing a wide array of intricate problems.
3. ADBO delivers impressive performance when tackling mixed problems, as indicated by the experimental outcomes spanning F11 to F20. In the 30-dimensional experiments, ADBO emerges as a clear frontrunner in functions such as F11, F15, F19, and F20, while maintaining competitive performance comparable to SSA in other functions. In the 100-dimensional experiments, ADBO outperforms its counterparts with remarkable efficiency in specific functions within F13, F15, F16, F19, and F20, establishing a significant lead. This remarkable performance can be attributed to its diverse solution search strategies. Its robust global search capabilities position it as a standout performer in addressing mixed problems.
4. In the realm of tackling composite problems, the experimental findings for ADBO concerning functions F21 through F29 undeniably underscore its compelling competitive edge. Within the domain of 30-dimensional experiments, ADBO showcases consistent superiority over alternative algorithms, with a notable surge in performance observed in the context of function F21. Transitioning to the 100-dimensional experiments, ADBO steadfastly maintains its supremacy in functions F21, F22, and F23 when compared to rival algorithms. These discoveries serve as a prominent testament to the distinctive advantages and adaptability inherent to ADBO for addressing composite problems. ADBO stands as an exceptional performer, solidifying its pivotal role in effectively navigating and optimizing intricate search spaces, making it an indispensable and potent tool for a diverse array of composite problems.

### 4.4. Wilcoxon Rank Sum Test

The non-parametric statistical test known as the Wilcoxon rank sum test was employed to assess whether the performance of the ADBO algorithm significantly distinguishes it from other algorithms. In this regard, results from 100 independent tests for each of the seven algorithms, conducted on the CEC2017 test functions, were used as the dataset. The Wilcoxon rank sum test was executed with a significance level of 0.05 to discern the presence of a statistically significant difference between the solution outcomes of the ADBO algorithm and the six comparative algorithms. Detailed test outcomes are documented in Table 5 and Table 7.

When *p* < 0.05, it indicates the rejection of the null hypothesis, signifying a significant difference between the two compared algorithms. Conversely, when *p* > 0.05, it suggests that these two algorithms yield comparable search outcomes. An examination of Table 5 and Table 7 clearly illustrates that the ADBO algorithm stands out significantly from the other algorithms. In summary, ADBO demonstrates a pronounced advantage when compared to SSA, HHO, BOA, OMA, WOA, SCA, and DBO, and this advantage is supported by strong statistical evidence.

**Table 5:** Wilcoxon rank sum test (Dim = 30).

	SSA	HHO	BOA	OMA	WOA	SCA	DBO
F1	3.02E-11 < 0.05	2.19E-08 < 0.05	2.95E-11 < 0.05	8.99E-11 < 0.05	1.34E-05 < 0.05	3.02E-11 < 0.05	4.11E-07 < 0.05
F2	3.99E-04 < 0.05	8.42E-01	3.34E-11 < 0.05	1.15E-01	3.02E-11 < 0.05	4.18E-09 < 0.05	1.41E-09 < 0.05
F3	8.20E-07 < 0.05	3.16E-10 < 0.05	3.02E-11 < 0.05	4.62E-10 < 0.05	5.01E-02	3.02E-11 < 0.05	4.42E-06 < 0.05
F4	4.03E-03 < 0.05	5.19E-07 < 0.05	3.02E-11 < 0.05	7.96E-01	6.79E-02	8.15E-11 < 0.05	5.97E-05 < 0.05
F5	1.30E-01	2.37E-10 < 0.05	3.02E-11 < 0.05	9.53E-07 < 0.05	3.33E-01 < 0.05	1.17E-09 < 0.05	1.77E-03 < 0.05
F6	1.11E-06 < 0.05	8.89E-10 < 0.05	3.02E-11 < 0.05	1.06E-03 < 0.05	7.24E-02	2.20E-07 < 0.05	3.51E-02 < 0.05
F7	3.64E-02 < 0.05	7.30E-04 < 0.05	3.02E-11 < 0.05	4.68E-02 < 0.05	6.20E-04 < 0.05	4.08E-11 < 0.05	2.28E-05 < 0.05
F8	5.20E-01	4.20E-10 < 0.05	3.02E-11 < 0.05	2.61E-02 < 0.05	1.22E-02 < 0.05	6.28E-06 < 0.05	1.44E-02 < 0.05
F9	6.28E-06 < 0.05	4.21E-02 < 0.05	3.02E-11 < 0.05	3.50E-09 < 0.05	1.16E-07 < 0.05	2.87E-10 < 0.05	9.35E-01
F10	2.16E-03 < 0.05	6.12E-10 < 0.05	3.02E-11 < 0.05	1.37E-01	4.03E-03 < 0.05	3.02E-11 < 0.05	1.31E-08 < 0.05
F11	2.39E-08 < 0.05	1.69E-09 < 0.05	3.02E-11 < 0.05	2.15E-02 < 0.05	1.04E-04 < 0.05	3.02E-11 < 0.05	8.56E-04 < 0.05
F12	5.75E-02	1.43E-08 < 0.05	3.02E-11 < 0.05	6.20E-01	2.25E-04 < 0.05	3.02E-11 < 0.05	8.48E-09 < 0.05
F13	1.49E-04 < 0.05	8.15E-05 < 0.05	3.02E-11 < 0.05	8.20E-07 < 0.05	8.15E-11 < 0.05	6.53E-08 < 0.05	6.00E-01
F14	6.35E-02	8.15E-11 < 0.05	3.02E-11 < 0.05	2.17E-01	6.20E-04 < 0.05	3.02E-11 < 0.05	6.01E-08 < 0.05
F15	5.55E-02	4.57E-09 < 0.05	3.02E-11 < 0.05	3.09E-06 < 0.05	1.27E-02 < 0.05	4.50E-11 < 0.05	3.59E-05 < 0.05
F16	6.41E-01	1.17E-02 < 0.05	3.69E-11 < 0.05	5.97E-05 < 0.05	1.44E-03 < 0.05	1.06E-03 < 0.05	3.39E-02 < 0.05
F17	4.64E-01	1.29E-06 < 0.05	3.02E-11 < 0.05	1.27E-02 < 0.05	1.73E-07 < 0.05	3.34E-11 < 0.05	1.44E-03 < 0.05
F18	3.27E-02	3.02E-11 < 0.05	3.02E-11 < 0.05	6.52E-01	1.44E-02 < 0.05	3.02E-11 < 0.05	4.31E-08 < 0.05
F19	5.27E-05 < 0.05	6.77E-05 < 0.05	3.34E-11 < 0.05	7.01E-02	1.33E-01	1.31E-08 < 0.05	6.10E-03 < 0.05
F20	9.47E-01	7.77E-09 < 0.05	3.02E-11 < 0.05	3.78E-02 < 0.05	6.97E-03 < 0.05	1.29E-09 < 0.05	5.27E-05 < 0.05
F21	2.07E-02 < 0.05	3.82E-09 < 0.05	4.98E-11 < 0.05	1.87E-05 < 0.05	1.50E-02 < 0.05	1.78E-10 < 0.05	3.01E-04 < 0.05
F22	1.70E-02 < 0.05	7.39E-11 < 0.05	3.69E-11 < 0.05	4.98E-04 < 0.05	4.73E-01	1.60E-07 < 0.05	9.33E-02
F23	5.19E-02	1.17E-09 < 0.05	1.46E-10 < 0.05	1.91E-01	3.63E-01	6.53E-07 < 0.05	1.91E-01
F24	8.99E-11 < 0.05	4.11E-07 < 0.05	3.02E-11 < 0.05	6.07E-11 < 0.05	7.06E-01	3.02E-11 < 0.05	7.98E-02
F25	5.01E-02 < 0.05	2.19E-08 < 0.05	4.98E-11 < 0.05	4.92E-01	5.49E-01	7.04E-07 < 0.05	4.64E-03 < 0.05
F26	1.78E-04 < 0.05	2.92E-09 < 0.05	3.02E-11 < 0.05	9.12E-01	3.95E-01	1.55E-09 < 0.05	9.47E-01
F27	1.61E-10 < 0.05	1.61E-06 < 0.05	3.02E-11 < 0.05	2.03E-09 < 0.05	4.12E-01	3.02E-11 < 0.05	2.13E-05 < 0.05
F28	8.31E-03 < 0.05	1.25E-05 < 0.05	3.02E-11 < 0.05	9.33E-02	6.41E-01	1.70E-08 < 0.05	8.24E-02
F29	2.15E-10 < 0.05	1.09E-10 < 0.05	3.02E-11 < 0.05	7.06E-01 < 0.05	2.15E-10 < 0.05	3.02E-11 < 0.05	3.83E-06 < 0.05

**Table 6:** Wilcoxon rank sum test (Dim = 100).

	SSA	HHO	BOA	OMA	WOA	SCA	DBO
F1	9.47E-01	3.02E-11 < 0.05	3.02E-11 < 0.05	3.02E-11 < 0.05	9.12E-01	3.02E-11 < 0.05	1.21E-10 < 0.05
F2	1.87E-05 < 0.05	2.24E-02 < 0.05	3.02E-11 < 0.05	4.94E-05 < 0.05	3.02E-11 < 0.05	5.00E-09 < 0.05	3.34E-11 < 0.05
F3	4.73E-01	2.03E-07 < 0.05	3.02E-11 < 0.05	2.03E-09 < 0.05	4.73E-01	3.02E-11 < 0.05	1.87E-05 < 0.05
F4	1.29E-06 < 0.05	6.01E-08 < 0.05	3.02E-11 < 0.05	2.17E-01	1.00E+00	1.21E-10 < 0.05	2.51E-02 < 0.05
F5	4.62E-10 < 0.05	3.34E-11 < 0.05	3.02E-11 < 0.05	9.76E-10 < 0.05	2.68E-06 < 0.05	4.50E-11 < 0.05	6.20E-01
F6	3.69E-11 < 0.05	3.02E-11 < 0.05	3.02E-11 < 0.05	3.01E-07 < 0.05	1.32E-04 < 0.05	3.02E-11 < 0.05	2.71E-01
F7	6.91E-04 < 0.05	1.22E-02 < 0.05	3.02E-11 < 0.05	6.10E-01	1.12E-02 < 0.05	8.99E-11 < 0.05	2.84E-04 < 0.05
F8	4.98E-11 < 0.05	4.08E-11 < 0.05	3.02E-11 < 0.05	1.39E-06 < 0.05	9.12E-01	6.07E-11 < 0.05	2.15E-06 < 0.05
F9	8.19E-01	1.17E-02 < 0.05	3.02E-11 < 0.05	4.08E-11 < 0.05	6.91E-04 < 0.05	3.02E-11 < 0.05	9.82E-01
F10	1.44E-03 < 0.05	3.26E-01	3.02E-11 < 0.05	3.67E-03 < 0.05	3.83E-05 < 0.05	3.02E-11 < 0.05	6.53E-08 < 0.05
F11	8.31E-03 < 0.05	1.21E-10 < 0.05	3.02E-11 < 0.05	2.53E-04 < 0.05	3.37E-05 < 0.05	3.02E-11 < 0.05	5.97E-09 < 0.05
F12	4.94E-05 < 0.05	1.36E-07 < 0.05	3.02E-11 < 0.05	9.47E-03 < 0.05	2.49E-06 < 0.05	3.02E-11 < 0.05	1.43E-05 < 0.05
F13	8.77E-02	5.09E-08 < 0.05	3.02E-11 < 0.05	3.34E-03 < 0.05	3.02E-11 < 0.05	8.99E-11 < 0.05	1.54E-01
F14	5.08E-03 < 0.05	2.20E-07 < 0.05	3.02E-11 < 0.05	1.05E-01	1.17E-05 < 0.05	3.02E-11 < 0.05	1.68E-04 < 0.05
F15	5.49E-01	3.77E-04 < 0.05	3.02E-11 < 0.05	1.12E-02 < 0.05	3.59E-05 < 0.05	1.09E-10 < 0.05	3.26E-01

**Table 7:** Wilcoxon rank sum test (Dim = 100).

	SSA	HHO	BOA	OMA	WOA	SCA	DBO
F16	3.18E-04 < 0.05	9.79E-05 < 0.05	3.02E-11 < 0.05	2.16E-03 < 0.05	2.42E-02 < 0.05	2.32E-06 < 0.05	7.62E-03 < 0.05
F17	4.36E-02 < 0.05	8.66E-05 < 0.05	3.02E-11 < 0.05	9.23E-01	2.03E-09 < 0.05	3.34E-11 < 0.05	2.89E-03 < 0.05
F18	2.58E-01	2.44E-09 < 0.05	3.02E-11 < 0.05	4.84E-02 < 0.05	1.99E-02 < 0.05	3.02E-11 < 0.05	3.34E-03 < 0.05
F19	2.71E-01	2.51E-02 < 0.05	3.02E-11 < 0.05	4.68E-02 < 0.05	2.28E-05 < 0.05	4.71E-04 < 0.05	9.93E-02
F20	5.11E-01	2.13E-05 < 0.05	3.02E-11 < 0.05	7.60E-07 < 0.05	8.88E-06 < 0.05	1.46E-10 < 0.05	8.42E-01
F21	3.67E-03 < 0.05	8.12E-04 < 0.05	4.50E-11 < 0.05	6.79E-02	1.37E-03 < 0.05	1.73E-07 < 0.05	4.23E-03 < 0.05
F22	1.81E-01	8.15E-11 < 0.05	3.02E-11 < 0.05	9.21E-05 < 0.05	2.12E-01	4.62E-10 < 0.05	5.30E-01
F23	1.68E-04 < 0.05	3.34E-11 < 0.05	3.02E-11 < 0.05	3.48E-01	1.67E-01	5.49E-11 < 0.05	5.55E-02
F24	3.63E-01	1.25E-07 < 0.05	3.02E-11 < 0.05	3.34E-11 < 0.05	2.42E-02 < 0.05	3.02E-11 < 0.05	1.47E-07 < 0.05
F25	1.54E-01	3.59E-05 < 0.05	3.02E-11 < 0.05	1.06E-03 < 0.05	3.33E-01	5.60E-07 < 0.05	2.97E-01
F26	3.18E-01	1.78E-10 < 0.05	3.02E-11 < 0.05	2.27E-03 < 0.05	3.16E-05 < 0.05	3.02E-11 < 0.05	1.81E-01
F27	2.23E-01	9.76E-10 < 0.05	3.02E-11 < 0.05	4.62E-10 < 0.05	2.58E-01	3.02E-11 < 0.05	2.03E-09 < 0.05
F28	4.86E-03 < 0.05	2.03E-09 < 0.05	3.02E-11 < 0.05	5.49E-01	2.77E-01	3.69E-11 < 0.05	7.06E-01
F29	2.89E-03 < 0.05	8.15E-11 < 0.05	3.02E-11 < 0.05	1.12E-01	1.34E-05 < 0.05	3.02E-11 < 0.05	5.56E-04 < 0.05

### 4.5. ADBO vs. Other Improved DBO Algorithms

**Figure 8:**
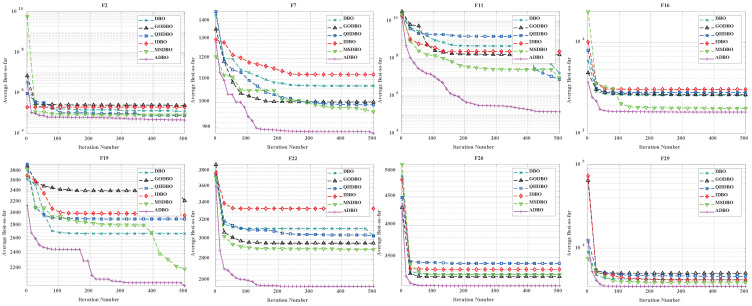
Multiple improved DBO vs. ADBO (Dim = 30).

**Figure 9:**
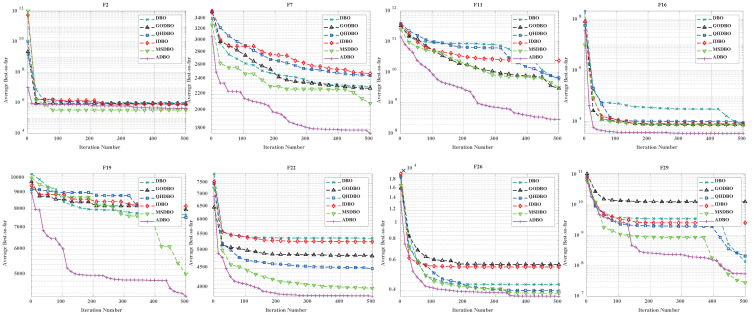
Multiple improved DBO vs. ADBO (Dim = 100).

To showcase the prowess of ADBO, we meticulously selected a spectrum of representative functions from CEC2017, including F2, F7, F11, F16, F19, F22, F26, and F29, and pitted ADBO against DBO, GODBO [29], QHDBO [9], IDBO [30], and MSDBO [26]. As depicted in Figure 8, the convergence curves vividly illustrate that when Dim = 30, F7, F11, F19, and F22 exhibit remarkable superiority over both DBO and its variants. Moreover, as illustrated in Figure 9, with Dim = 100, the performance of F7, F11, F16, F19, F22, and F29 significantly outshines that of DBO and its variants. These findings compellingly affirm the exceptional efficacy of the ADBO algorithm.

## 5. Engineering Optimization Issues

### 5.1. Optimization of Robotic Gripper Performance

**Figure 10:**
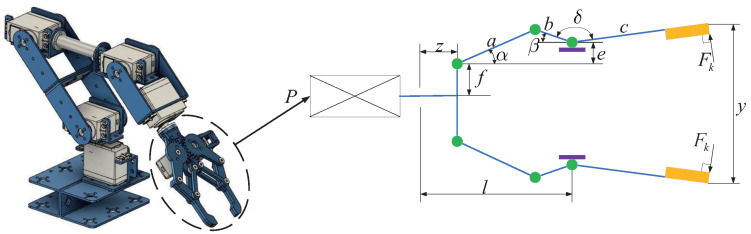
Mechanical arm image.

The challenge posed by the robotic gripper problem, as referenced in [31], presents a multifaceted and pivotal conundrum within the domain of mechanical structure engineering, as visually represented in Figure 10. The resolution of this issue holds paramount importance for achieving the efficiency of robotic gripping and manipulation. This problem delves into the consideration of six critical factors: link length, the angular relationship between the links, vertical displacement, clamping pressure, actuator displacement of the robotic gripper, and horizontal displacement.

To begin with, the lengths of the links (*a*, *b*, *c*) play a crucial role in determining the stability and operational range of the robotic gripper. Variations in link lengths can significantly affect the gripper’s flexibility and adaptability, necessitating the careful optimization of these parameters.

Furthermore, the geometric angle (*d*) represents another crucial factor, determining the relative positions and angles among the gripper’s components, thereby influencing gripping efficiency and precision. Vertical displacement (*e*) pertains to the gripper’s ability to move vertically, a critical feature for handling objects of various sizes and shapes. Effective control of vertical displacement enhances the gripper’s adaptability.

The clamping pressure represents the force exerted by the gripper to secure the grasped object. This parameter directly affects the gripper’s grasping capability and stability. Fine-tuning and optimizing the magnitude of the clamping force are necessary to suit specific applications.

Actuator displacement (*f*) and horizontal displacement (*l*) of the robotic gripper represent the vertical and horizontal distances between the actuator end and the link node. These parameters intricately influence the gripper’s range of motion and adaptability.

To effectively tackle this intricate challenge, we have incorporated seven optimization variables ($x_{1}$ to $x_{7}$), with each variable aligning with the aforementioned factors. By skillfully optimizing these variables, the robotic gripper can attain peak performance across a spectrum of tasks and environmental conditions. The comprehensive mathematical model, which includes these seven variables and their corresponding constraints, is outlined below, providing invaluable insights for the design and optimization of robotic grippers. Through adept engineering design and mathematical modeling, we can attain efficiency, precision, and adaptability in robot gripping operations, thereby unleashing significant potential in the realm of automation.

Consider the following variable:

(14) $$\begin{array}{c} x=\left(x_{1},x_{2},x_{3},x_{4},x_{5},x_{6},x_{7}\right)=\left(a,b,c,e,f,l,\delta \right) \end{array}$$

Minimize

(15) $$\begin{array}{c} f\left(x\right)=-\min_{z}F_{k}\left(x,z\right)+\max_{z}F_{k}\left(x,z\right) \end{array}$$

Subject to

(16) $$\begin{array}{cc} \; & g_{1}\left(x\right)=-Y_{\min}+y\left(\left(x\right),Z_{\max}\right)\leq 0\\ \; & g_{2}\left(x\right)=-y\left(\left(x\right),Z_{\max}\right)\leq 0\\ \; & g_{3}\left(x\right)=Y_{\max}-y\left(\left(x\right),0\right)\leq 0\\ \; & g_{4}\left(x\right)=y\left(\left(x\right),0\right)-Y_{G}\leq 0\\ \; & g_{5}\left(x\right)=l^{2}+e^{2}-{\left(a+b\right)}^{2}\leq 0\\ \; & g_{6}\left(x\right)=b^{2}-{\left(a-e\right)}^{2}-{\left(l-Z_{\max}\right)}^{2}\leq 0\\ \; & g_{7}\left(x\right)=Z_{\max}-l\leq 0 \end{array}$$

where $\alpha =\cos_{-1}\left(\frac{a^{2}+g^{2}-b^{2}}{2ag}\right)+\phi$, $g=\sqrt{e^{2}+{\left(z-l\right)}^{2}}$, $\beta =\cos_{-1}\left(\frac{g^{2}+b^{2}-a^{2}}{2ag}\right)-\phi$, $\phi =\tan_{-1}\left(\frac{e}{1-z}\right),y\left(x,z\right)=2\left(f+e+c\cdot \sin\left(\beta +\delta \right)\right)$, $F_{k}=\frac{Pb\sin(\alpha +\beta )}{2c\cdot \cos(\alpha )},Y_{\min}=50,Y_{\max}=100,Y_{G}=150,Z_{\max}=100,P=100$.

With bounds, the following is obtained: 0≤e≤50,100≤c≤200,10≤f,a,b≤150,1≤δ≤3.14,100≤l≤300.

**Figure 11:**
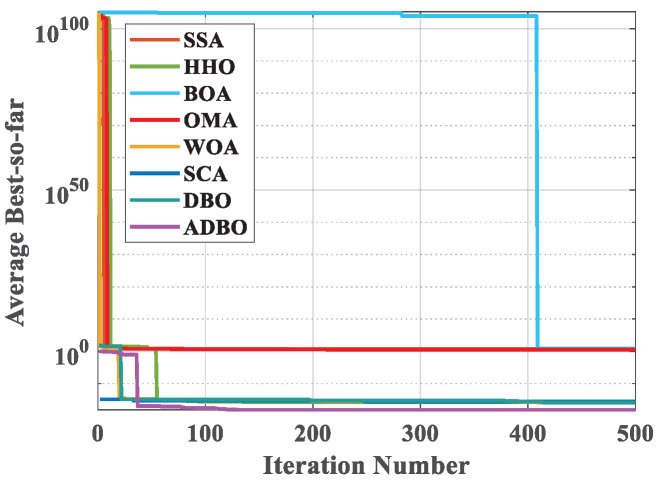
Mechanical arm convergence plot.

The primary objective of the robot gripper problem is to optimize the disparity between the maximum and minimum forces produced by the robot gripper, which is crucial for ensuring the stability and precision of robot gripping operations. In Table 8, we meticulously present the numerical results of the ADBO algorithm and other competing algorithms as they tackle this challenge. Upon examining Figure 11, the convergence of the ADBO algorithm becomes readily apparent, and the results unequivocally demonstrate its superior search performance, surpassing all other algorithms. Table 9 provides statistical data derived from 100 independent experiment repetitions on the mean, variance, minimum, and maximum values of the minimum force. It is evident that the ADBO algorithm consistently achieves the lowest mean force.

The ADBO algorithm furnishes optimal values for the variables, specifically x∗=(100,38.2,200,0,19.6,100,1.61) accompanied by a corresponding fitness value of $f\left(x*\right)\;=\;6.54\;E-19$. This signifies that the ADBO algorithm has effectively identified a highly optimized solution with virtually no remaining error, an aspect of paramount significance for the stability and performance of the gripper.

### 5.2. Three-Bar Truss Design Problem

**Figure 12:**
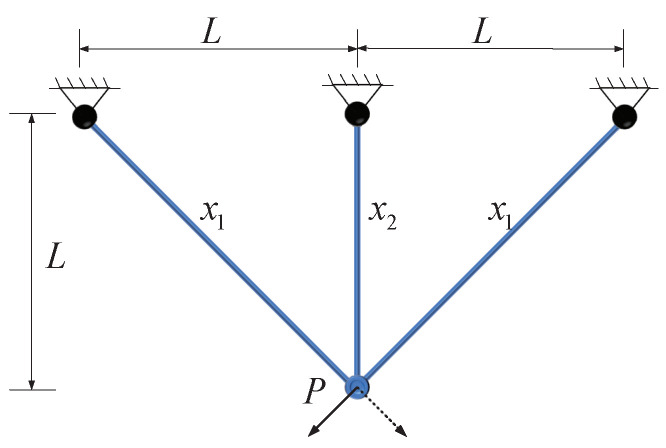
Triangle truss design.

The challenge of designing a truss with three poles is a significant engineering task. This problem, originally posed by Nowacki, revolves around the objective of minimizing the truss’s overall volume while ensuring that the stress on each side of every truss member remains within predefined limits. It stands as a pivotal engineering optimization conundrum because, in the realm of engineering design, there is a perpetual need to create structures that are both structurally robust and resource-efficient, thereby minimizing material usage and waste. The truss with three poles in the design is illustrated in Figure 12.

Within this problem, a delicate equilibrium must be established between two fundamental considerations. Firstly, it is imperative to ensure that each component of the truss can withstand the applied stresses as dictated by the design parameters, thereby guaranteeing the safety and stability of the structure. Secondly, it is of utmost importance to reduce the overall volume of the truss to optimize material costs and minimize the structure’s weight. This is crucial across a spectrum of engineering projects, including construction, aerospace, and various other domains.

Solving this challenge requires intricate mathematical modeling and optimization analysis. Researchers must consider a multitude of factors, including the truss’s geometric configuration, material characteristics, and stress distribution, among others, to determine the most suitable design solution. Typically, addressing this kind of dilemma involves a combination of engineering and mathematical techniques, such as linear programming, nonlinear programming, and structural optimization, to find the optimal truss design that meets stress criteria while minimizing volume. This holds significant practical importance in contemporary engineering and architectural fields as it assists in creating structures that are not only more efficient but also cost-effective.

Consider the variable

(17) $$\begin{array}{c} x=(x_{1},x_{2}). \end{array}$$

Minimize

(18) $$\begin{array}{c} \min f\left(x\right)=\left(2\sqrt{2}x_{1}+x_{2}\right)\times L. \end{array}$$

Subject to

(19) $$\begin{array}{cc} \; & g_{1}=\frac{\sqrt{2}x_{1}+x_{2}}{\sqrt{2}x_{1}^{2}+2x_{1}x_{2}}P-\sigma \leq 0\\ \; & g_{2}=\frac{x_{2}}{\sqrt{2}x_{1}^{2}+2x_{1}x_{2}}P-\sigma \leq 0\\ \; & g_{3}=\frac{1}{x_{1}+\sqrt{2}x_{2}}P-\sigma \leq 0 \end{array}$$

where P=2,L=100,σ=2.

With bounds, the following is obtained: $0\leq x_{1},x_{2}\leq 2$.

The experimental results of the eight algorithms applied to the three-rod truss design problem are summarized in Table 10. It is clear from the table that, in comparison to the reference algorithm, the ADBO algorithm outperforms the others, demonstrating the highest level of performance.

**Figure 13:**
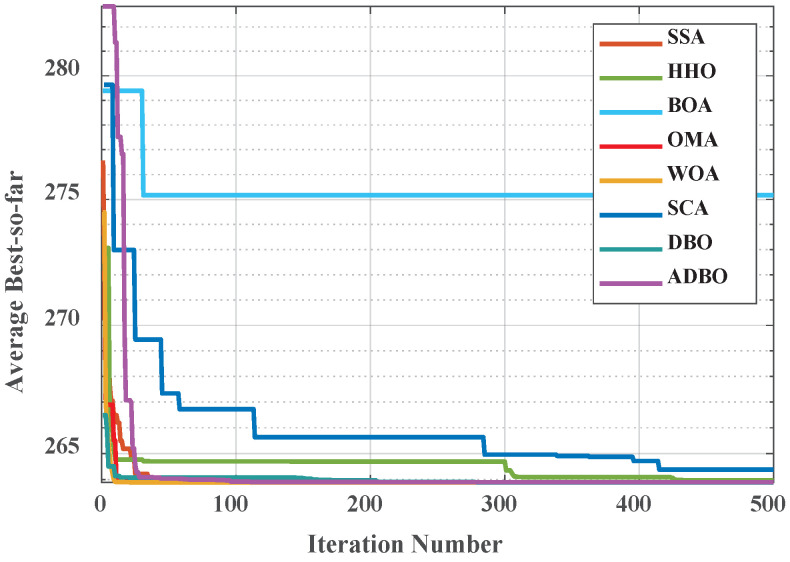
Three-bar truss convergence curve diagram.

This highlights the proficiency of the ADBO algorithm in finding optimal solutions for the three-rod truss design problem, showcasing superior efficiency and accuracy. Upon examining Figure 13, the convergence of the ADBO algorithm becomes evident, unequivocally demonstrating its superior search performance over all other algorithms. Additionally, Table 11 presents the statistical results from 100 independent repetitions of the ADBO algorithm, confirming its ability to achieve the optimal mean value.

This outcome carries significant implications for the field of engineering design and optimization, suggesting that the ADBO algorithm holds promise as the preferred choice. It aids engineers and researchers in more effectively designing and optimizing complex truss structures, meeting stress constraints while simultaneously reducing volume and costs. This capability contributes to enhancing engineering design across various application domains, including construction, aerospace, and numerous other engineering projects. Hence, these research findings robustly support future engineering practices.

### 5.3. Unmanned Aerial Vehicle Path Planning

In the context of this research, the path planning problem is articulated through a cost function that incorporates both optimal criteria and drone constraints, as detailed below:

To ensure the optimal efficiency of unmanned aerial vehicle (UAV) operations, the planned paths must meet specific standards tailored to the application’s requirements [32]. Given our focus on aerial photography, mapping, and surface inspection, our optimization criterion is to minimize the path length. Since UAVs are controlled through ground control stations, the flight path (denoted as $X_{i}$) is expressed as a sequence of *n* waypoints. Each waypoint corresponds to a path node in the search map and is characterized by coordinates denoted as $P_{ij}=\left(x_{ij},y_{ij},z_{ij}\right)$. The cost $F_{1}$ associated with the path length is calculated using the formula $F_{1}\left(X_{i}\right)=\sum_{j=1}^{n-1}∥P_{ij}{\vec{P}}_{i,j+1}∥$, where $∥P_{ij}{\vec{P}}_{i,j+1}∥$ represents the Euclidean distance between two nodes.

**Figure 14:**
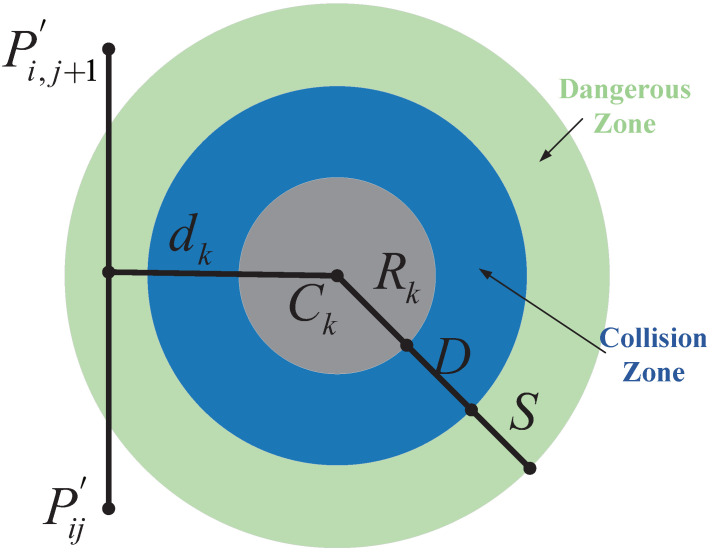
Threat cost.

In addition to pursuing optimality, the planned path must also ensure the safe operation of the UAV by guiding it around obstacles commonly encountered in the operational space. Let *K* represent the set of all threats, where each threat is defined within a cylindrical region with a projected center coordinate $C_{k}$ and radius $R_{k}$, as illustrated in Figure 14. For a given path segment $∥P_{ij}{\vec{P}}_{i,j+1}∥$, the associated threat cost is proportional to the distance $d_{k}$ between the UAV and the threat center coordinate $C_{k}$. By taking into account the diameter *D* of the threat region, the UAV’s safety distance *S*, and the distance to the collision zone, the threat cost $F_{2}$ is calculated across waypoints on the path segment $P_{ij}$ for the obstacle set *K*, as follows:

(20) $$\begin{array}{c} \left\{\begin{array}{c} F_{2}\left(X_{i}\right)=\sum_{j=1}^{n-1}\sum_{k=1}^{K}T_{k}\left(P_{ij}{\vec{P}}_{i,j+1}\right),\\ T_{k}\left(P_{ij}{\vec{P}}_{i,j+1}\right)=\left\{\begin{array}{cc} 0, & \;\mathrm{if}\;d_{k}>S+D+R_{k}\\ \left(S+D+R_{k}\right)-d_{k}, & \;\mathrm{if}\;D+R_{k}<d_{k}\leq S+D+R_{k}\\ \infty , & \;\mathrm{if}\;d_{k}\leq D+R_{k} \end{array}\right. \end{array}\right. \end{array}$$

During operations, it is customary to confine the flight altitude within specified limits, specifically the minimum and maximum heights. For example, in measurement and search applications, the camera is required to capture data at a predefined resolution and field of view, resulting in constraints on the flight altitude. Let $h_{\min}$ and $h_{\max}$ represent the minimum and maximum heights, respectively. The elevation cost $P_{ij}$ associated with waypoints is then calculated using the following formula:

(21) $$\begin{array}{c} H_{ij}=\left\{\begin{array}{cc} \left|h_{ij}-\frac{\left(h_{\max}+h_{\min}\right)}{2}\right|, & \;\mathrm{if}\;h_{\min}\leq h_{ij}\leq h_{\max}\\ \infty , & \;\mathrm{otherwise},\; \end{array}\right. \end{array}$$

**Figure 15:**
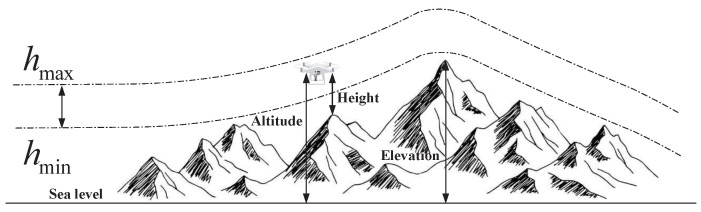
Elevation cost.

In this context, *h* represents the flight altitude relative to the ground, as depicted in Figure 15. Notably, *H* maintains an average altitude while penalizing values that exceed the specified range. Summarizing *H* across all waypoints provides the altitude cost, as follows:

(22) $$\begin{array}{c} F_{3}\left(X_{i}\right)=\sum_{j=1}^{n}H_{ij} \end{array}$$

**Figure 16:**
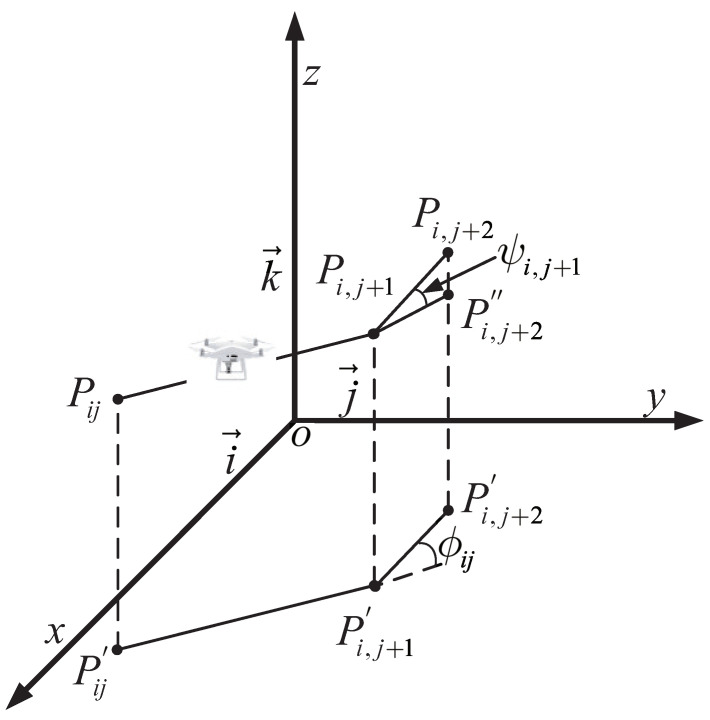
Turn angle and climb angle description.

The smoothness cost evaluates turning and climb rates, crucial for generating viable paths. As depicted in Figure 16, the turning angle $\phi_{ij}$ between consecutive path segments, $P_{ij}^{\prime }{\vec{P}}_{i,j+1}^{\prime }$ and $P_{i,j+1}^{\prime }{\vec{P}}_{i,j+2}^{\prime }$, is projected onto the horizontal plane Oxy. Let $\vec{k}$ represent the unit vector along the *z*-axis direction, and the projection vector can be calculated as

(23) $$\begin{array}{c} F_{3}\left(X_{i}\right)=\sum_{j=1}^{n}H_{ij}P_{ij}^{\prime }{\vec{P}}_{i,j+1}^{\prime }=\vec{k}\times \left(P_{ij}{\vec{P}}_{i,j+1}\times \vec{k}\right) \end{array}$$

Therefore, the formula for calculating the turning angle is

(24) $$\begin{array}{c} \phi_{ij}=\arctan\left(\frac{\left\|P_{ij}^{\prime }{\vec{P}}_{i,j+1}^{\prime }\times P_{i,j+1}^{\prime }{\vec{P}}_{i,j+2}^{\prime }\right\|}{P_{ij}^{\prime }{\vec{P}}_{i,j+1}^{\prime }\cdot P_{i,j+1}^{\prime }{\vec{P}}_{i,j+2}^{\prime }}\right) \end{array}$$

The climb angle, denoted as $\varphi_{ij}$, represents the angle between path segments, considering both $P_{ij}{\vec{P}}_{i,j+1}$ and its projection, $P_{ij}^{\prime }{\vec{P}}_{i,j+1}^{\prime }$, onto the horizontal plane. It is determined by the following formula:

(25) $$\begin{array}{c} \psi_{ij}=\arctan\left(\frac{z_{i,j+1}-z_{ij}}{\left\|\vec{P}P_{i,j+1}^{\prime }\right\|}\right) \end{array}$$

Then, the formula for calculating the smoothness cost is

(26) $$\begin{array}{c} F_{4}\left(X_{i}\right)=a_{1}\sum_{j=1}^{n-2}\phi_{ij}+a_{2}\sum_{j=1}^{n-1}\left|\psi_{ij}-\psi_{i,j-1}\right| \end{array}$$

Here, $a_{1}$ and $a_{2}$ are the penalty coefficients for the turning angle and climb angle, respectively.

By considering optimality, safety, and feasibility constraints associated with the path, the total cost function can be defined in the following form:

(27) $$\begin{array}{c} F\left(X_{i}\right)=\sum_{k=1}^{4}b_{k}F_{k}\left(X_{i}\right) \end{array}$$

where represents the weight coefficients. The costs are associated with path length, threat, smoothness, and flight altitude, respectively. The decision variables include a list of waypoints such that, where is the operational space of the UAV. With these definitions, the cost function is fully determined and can be utilized as input for the path planning process.

**Figure 17:**
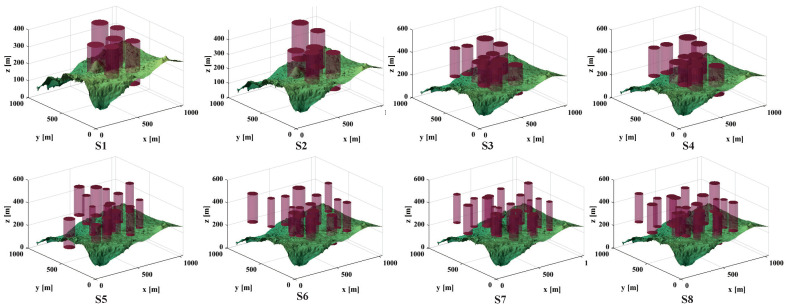
UAV scenarios.

**Figure 18:**
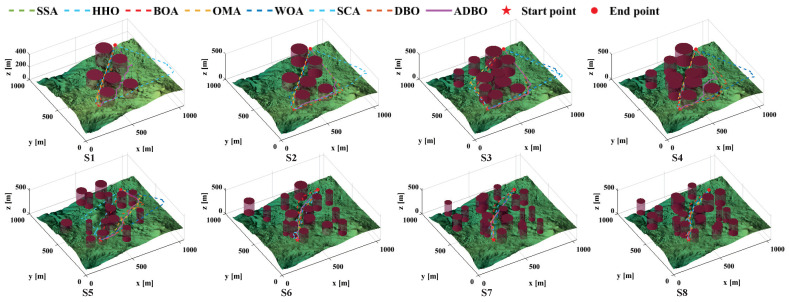
Paths in UAV scenarios.

**Figure 19:**
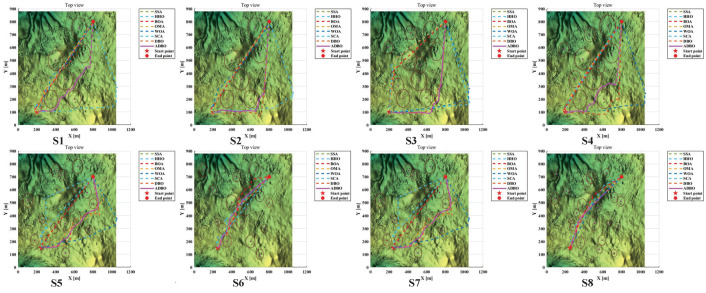
Overhead perspective of the UAV path.

**Figure 20:**
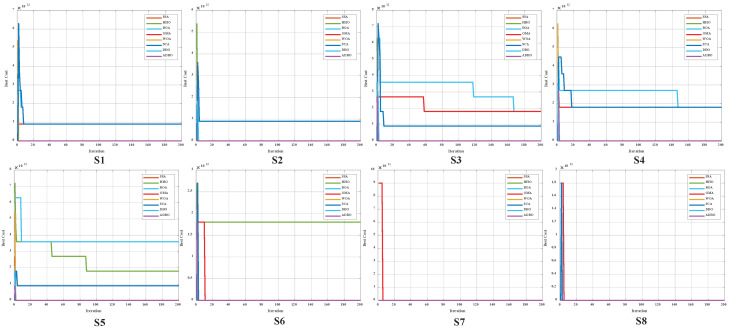
Iteration graph of the UAV path.

Based on the examples provided, we conducted tests across eight distinct scenarios, as illustrated in Figure 17. The corresponding planned paths for these scenarios are shown in Figure 18, with top views presented in Figure 19, and the convergence iteration graph displayed in Figure 20. It is noteworthy that under conditions of moderate obstacle complexity, the ADBO algorithm outperforms other algorithms, highlighting its effectiveness in navigating challenging environments. Additionally, the ADBO algorithm demonstrates robust performance in path planning across a variety of scenarios, showcasing its versatility.

To further validate these findings, we independently replicated the experiments 100 times, gathering data on averages, variances, and minimum values. The statistical results from these 100 experiment repetitions, summarized in Table 12, reveal that the ADBO algorithm excels in these performance metrics, underscoring its stability and reliability. Specifically, in the eight scenarios involving unmanned aerial vehicles (UAVs), the ADBO algorithm consistently ranks first in terms of average values. This notable achievement not only underscores its superiority in path planning but also highlights its potential to significantly reduce UAV energy consumption while enhancing safety measures. Further analysis reveals that the ADBO algorithm quickly converges to the optimal solution in scenarios S1–S5, demonstrating its advantage in situations requiring rapid algorithmic speed, such as UAV applications. This consistent performance further establishes the ADBO algorithm as a leading solution in the field of path planning.

An in-depth examination of these results indicates that the ADBO algorithm not only achieves impressive outcomes in individual experiments but also maintains consistently superior performance across a variety of experimental conditions. This consistency suggests that the ADBO algorithm possesses high levels of stability and reliability when tackling path planning challenges in complex scenarios, providing strong support for practical applications.

## 6. Conclusions

To improve the performance and flexibility of the original DBO algorithm, this study presents three significant enhancements. First, we introduce a Gaussian chaotic strategy to infuse greater randomness into the algorithm, which facilitates a more thorough exploration of the solution space and enhances its global search capabilities. Second, we integrate a spiral search strategy that uses a spiral trajectory to balance global exploration and local exploitation, thus optimizing the overall performance of the algorithm. Third, we adopt an adaptive convergence factor to better synchronize the algorithm’s search strategies in both the early and later stages, thereby increasing its robustness and overall efficiency.

To assess the effectiveness of the enhanced ADBO algorithm, we conducted a comprehensive evaluation using the CEC2017 benchmark functions. The results indicate that the improved ADBO algorithm significantly boosts global search capacity in the early stages, reducing the likelihood of becoming trapped in local optima, while maintaining high iteration speed in later stages, thus enhancing local exploitation efficiency. These improvements clearly demonstrate the effectiveness of the proposed enhancements.

In addressing engineering problems, we considered three classic cases: robot manipulator design, a triangular linkage problem, and UAV path planning. Specifically, for UAVs, the ADBO algorithm showed notable improvements in path planning tasks. It not only enhances the safety of drones by effectively avoiding obstacles but also reduces energy consumption, thereby increasing the efficiency of path planning. These outcomes suggest that the ADBO algorithm has significant potential in engineering applications, particularly in practical scenarios like wireless sensor network coverage.

Future research directions could involve further optimization of algorithm parameters to adapt to more complex engineering challenges. Investigating the application of the algorithm in large-scale systems and combining it with other optimization techniques could enhance its adaptability and robustness. Additionally, a deeper exploration into the biological inspirations behind the algorithm to extract more advantageous features represents a promising direction for future research. These efforts will help expand the application scope of the ADBO algorithm and advance the development of swarm intelligence techniques for solving real-world engineering problems.

## Figures and Tables

**Table 1:** CEC2017 functions.

Type	No.	Function	Minimum Value
Unimodal functions	1	Shifted and Rotated Bent Cigar Function	100
	2	Shifted and Rotated Zakharov Function	200
	3	Shifted and Rotated Rosenbrock’s Function	300
	4	Shifted and Rotated Rastrigin’s Function	400
	5	Shifted and Rotated Expanded Scaffer’s F6 Function	500
Simple multimodal functions	6	Shifted and Rotated Lunacek Bi_Rastrigin Function	600
	7	Shifted and Rotated Non-Continuous Rastrigin’s Function	700
	8	Shifted and Rotated Levy Function	800
	9	Shifted and Rotated Schwefel’s Function	900
Hybrid functions	10	Hybrid Function 1 (N=3)	1000
	11	Hybrid Function 2 (N = 3)	1100
	12	Hybrid Function 3 (N = 3)	1200
	13	Hybrid Function 4 (N = 4)	1300
	14	Hybrid Function 5 (N = 4)	1400
	15	Hybrid Function 6 (N = 4)	1500
	16	Hybrid Function 6 (N = 5)	1600
	17	Hybrid Function 6 (N = 5)	1700
	18	Hybrid Function 6 (N = 5)	1800
	19	Hybrid Function 6 (N = 6)	1900
Composition functions	20	Composition Function 1 (N = 3)	2000
	21	Composition Function 2 (N = 3)	2100
	22	Composition Function 3 (N = 4)	2200
	23	Composition Function 4 (N = 4)	2300
	24	Composition Function 5 (N = 5)	2400
	25	Composition Function 6 (N = 5)	2500
	26	Composition Function 7 (N = 6)	2600
	27	Composition Function 7 (N = 6)	2700
	28	Composition Function 9 (N = 3)	2800
	29	Composition Function 10 (N = 3)	2900
Search range: ${\left[-100,100\right]}^{D}$			

**Table 2:** Algorithm parameters.

Algorithm	Population	Size Number of Iterations	Parameters
SSA	30	500	PD=0.2;SD=0.1;R2=0.8
HHO	30	500	β=1.5;r=0.5;E=0.5
BOA	30	500	P=0.8;pe=0.1;sm=0.01
OMA	30	500	NA=1.40
WOA	30	500	$a=2*(1-t/T_{\max});k=1$
SCA	30	500	a=2
DBO	30	500	RDB=6;EDB=6;FDB=7;SDB=11
ADBO	30	500	RDB=6;EDB=6;FDB=7;SDB=11; $\omega_{\max}=0.904;\omega_{\min}=0.782$

**Table 8:** Robotic arm parameters. Bold text indicates the optimal values.

Algorithms	Optimum Variables							Force Difference	Ranking
	$x_{1}\left(a\right)$	$x_{2}\left(b\right)$	$x_{3}\left(c\right)$	$x_{4}\left(e\right)$	$x_{5}\left(f\right)$	$x_{6}\left(l\right)$	$x_{7}\left(\delta \right)$		
SSA	1.50E+02	1.31E+02	1.00E+02	1.92E+01	3.38E+01	1.00E+02	1.97E+00	5.35E+00	7
HHO	9.95E+01	3.77E+01	1.01E+02	0.00E+00	1.00E+01	1.00E+02	1.20E+00	1.51E-16	4
BOA	1.50E+02	1.50E+02	1.00E+02	0.00E+00	1.50E+02	1.00E+02	3.14E+00	8.58E+00	8
OMA	1.49E+02	1.42E+02	2.09E+02	6.35E+00	1.76E+02	1.29E+02	2.66E+00	3.33E+00	6
WOA	1.00E+02	3.82E+01	1.00E+02	0.00E+00	1.03E+01	1.00E+02	1.08E+00	1.45E-16	3
SCA	9.10E+01	2.56E+01	1.60E+02	0.00E+00	1.81E+01	1.00E+02	1.75E+00	2.54E-16	5
DBO	9.37E+01	3.19E+01	2.00E+02	0.00E+00	1.00E+01	1.00E+02	1.70E+00	1.19E-16	2
ADBO	1.00E+02	3.82E+01	2.00E+02	0.00E+00	1.96E+01	1.00E+02	1.61E+00	**6.54E-19**	1

**Table 9:** Statistical measurement analysis of robotic arm clamping force. Bold text indicates the optimal values.

	SSA	HHO	BOA	OMA	WOA	SCA	DBO	ADBO
Mean	2.90E+00	1.11E+01	2.64E+104	3.67E+00	9.20E-02	2.29E-16	1.81E-16	**1.36E-16**
Std	4.12E+00	3.99E+02	2.19E+209	5.02E-01	2.54E-01	7.14E-33	1.80E-32	5.62E+00
Min	7.27E-17	1.61E-16	8.58E+00	4.78E-01	7.27E-17	9.03E-17	7.27E-17	4.37E-19
Max	6.67E+00	7.91E+01	2.19E+105	4.53E+00	2.76E+00	4.97E-16	5.43E-16	6.90E+00

**Table 10:** Cantilever beam design issues.

Algorithms	Optimum Variables		Best Value	Ranking
	Variable 1	Variable 2		
SSA	0.76273	0.47885	264.3961	7
HHO	0.7771	0.44198	263.9982	3
BOA	0.76705	0.45301	264.2257	5
OMA	0.78825	0.40944	263.8959	2
WOA	0.76578	0.4768	264.3125	6
SCA	0.77043	0.46517	264.4205	8
DBO	0.77599	0.44236	264.0209	4
ADBO	0.78867	0.40824	**263.8958**	1

**Table 11:** Three-bar truss design statistics. Bold text indicates the optimal values.

	SSA	HHO	BOA	OMA	WOA	SCA	DBO	ADBO
Mean	264.1369	264.0372	274.8986	263.8959	263.9758	268.5568	263.8994	**263.8959**
Std	1.53E-06	2.99E-02	4.09E+01	2.79E-08	6.69E-08	6.43E+01	1.86E-05	1.96E-04
Min	263.8958	263.8963	265.7236	263.8958	263.8958	263.964	263.8958	263.8958
Max	264.4213	264.6211	282.8427	263.8967	264.4157	282.8425	264.1727	263.9175

**Table 12:** Statistical table of 100 independent repetitions in UAV path planning. Bold text indicates the optimal values. Bold text indicates the optimal values.

		SSA	HHO	BOA	OMA	WOA	SCA	DBO	ADBO
Scene1	Mean	1.80E+11	7.20E+11	8.40E+11	8.40E+11	1.35E+04	7.80E+11	1.35E+04	**1.33E+04**
	Std	1.34E+23	1.34E+23	5.21E+22	5.21E+22	3.39E+06	9.68E+22	2.84E+06	1.64E+06
	Min	1.07E+04	1.15E+04	1.70E+04	9.44E+03	8.61E+03	1.65E+04	1.05E+04	1.10E+04
	Ranking	4	5	8	7	2	6	3	1
Scene2	Mean	1.80E+11	6.30E+11	9.00E+11	9.00E+11	1.30E+04	8.10E+11	1.34E+04	**1.26E+04**
	Std	1.34E+23	1.76E+23	7.05E+04	6.57E+02	4.01E+06	7.54E+22	1.33E+06	1.74E+06
	Min	1.07E+04	8.73E+03	9.00E+11	9.00E+11	8.23E+03	1.28E+04	1.18E+04	9.91E+03
	Ranking	4	5	8	7	2	6	3	1
Scene3	Mean	3.00E+10	7.59E+03	4.50E+11	7.06E+03	6.96E+03	1.06E+04	3.00E+10	**6.75E+03**
	Std	2.70E+22	2.71E+05	2.09E+23	7.65E+03	2.10E+03	2.40E+06	2.70E+22	2.70E+07
	Min	7.21E+03	7.05E+03	8.45E+03	6.91E+03	6.84E+03	8.30E+03	7.27E+03	6.57E+03
	Ranking	6	4	8	3	2	5	7	1
Scene4	Mean	3.00E+11	1.44E+12	1.80E+12	1.80E+12	1.50E+04	1.80E+12	1.44E+04	**1.32E+04**
	Std	4.66E+23	5.36E+23	3.28E+05	9.56E+02	1.25E+06	1.37E+07	2.57E+06	1.48E+06
	Min	1.18E+04	1.37E+04	1.80E+12	1.80E+12	1.29E+04	1.80E+12	1.16E+04	1.13E+04
	Ranking	4	5	7	6	3	8	2	1
Scene5	Mean	3.00E+11	6.90E+11	1.14E+12	3.30E+11	3.00E+10	3.90E+11	1.14E+04	**9.94E+03**
	Std	1.86E+23	1.50E+23	2.76E+23	1.95E+23	2.70E+22	2.06E+23	1.64E+06	2.75E+06
	Min	7.63E+03	1.01E+04	1.17E+04	7.11E+03	6.83E+03	9.64E+03	8.89E+03	8.07E+03
	Ranking	4	7	8	5	3	6	2	1
Scene6	Mean	7.00E+03	8.67E+03	9.00E+10	6.73E+03	6.72E+03	8.56E+03	8.26E+03	**6.69E+03**
	Std	3.38E+04	1.27E+06	7.54E+22	1.34E+04	1.93E+04	7.12E+05	5.80E+05	1.71E+02
	Min	6.66E+03	6.67E+03	8.52E+03	6.56E+03	6.55E+03	7.10E+03	6.99E+03	6.59E+03
	Ranking	4	7	8	3	2	6	5	1
Scene7	Mean	6.97E+03	7.96E+03	6.00E+10	6.72E+03	6.65E+03	7.89E+03	7.71E+03	**6.62E+03**
	Std	1.49E+04	1.07E+06	5.21E+22	5.98E+03	4.87E+03	1.99E+05	3.09E+05	7.89E+04
	Min	6.77E+03	6.77E+03	9.27E+03	6.57E+03	6.55E+03	7.19E+03	6.80E+03	6.58E+03
	Ranking	4	7	8	3	2	6	5	1
Scene8	Mean	6.00E+10	3.60E+11	5.10E+11	6.75E+03	6.88E+03	3.00E+10	9.02E+03	**6.69E+03**
	Std	1.08E+23	5.36E+23	7.09E+23	6.21E+03	2.42E+04	2.70E+22	5.79E+05	2.71E+04
	Min	6.86E+03	7.07E+03	9.43E+03	6.63E+03	6.65E+03	7.87E+03	7.61E+03	6.60E+03
	Ranking	6	7	8	2	3	5	4	1

## Data Availability

The original contributions presented in the study are included in the article, further inquiries can be directed to the corresponding authors.
